# Analysis of the role of thrombomodulin in all-trans retinoic acid treatment of coagulation disorders in cancer patients

**DOI:** 10.1186/s12976-019-0099-z

**Published:** 2019-02-14

**Authors:** Hamed Ghaffari, Jeffrey D. Varner, Linda R. Petzold

**Affiliations:** 1 Department of Mechanical Engineering, University of California Santa Barbara, Santa Barbara, CA 93106 USA; 2 School of Chemical and Biomolecular Engineering, Cornell University, Ithaca, NY 14853 USA; 3 Department of Computer Science, University of California Santa Barbara, Santa Barbara, CA 93106 USA

**Keywords:** All-trans retinoic acid, Thrombomodulin, Mathematical model, Acute promyelocytic leukemia, Disseminated intravascular coagulation, Pharmacokinetics, Pharmacodynamics

## Abstract

**Background:**

Clinical studies have shown that all-trans retinoic acid (RA), which is often used in treatment of cancer patients, improves hemostatic parameters and bleeding complications such as disseminated intravascular coagulation (DIC). However, the mechanisms underlying this improvement have yet to be elucidated. In vitro studies have reported that RA upregulates thrombomodulin (TM) expression on the endothelial cell surface. The objective of this study was to investigate how and to what extent the TM concentration changes after RA treatment in cancer patients, and how this variation influences the blood coagulation cascade.

**Results:**

In this study, we introduced an ordinary differential equation (ODE) model of gene expression for the RA-induced upregulation of TM concentration. Coupling the gene expression model with a two-compartment pharmacokinetic model of RA, we obtained the time-dependent changes in TM and thrombomodulin-mRNA (TMR) concentrations following oral administration of RA. Our results indicated that the TM concentration reached its peak level almost 14 h after taking a single oral dose (110 $\frac{mg}{m^2}$) of RA. Continuous treatment with RA resulted in oscillatory expression of TM on the endothelial cell surface. We then coupled the gene expression model with a mechanistic model of the coagulation cascade, and showed that the elevated levels of TM over the course of RA therapy with a single daily oral dose (110 $\frac{mg}{m^2}$) of RA, reduced the peak thrombin levels and endogenous thrombin potential (ETP) up to 50 and 49%, respectively. We showed that progressive reductions in plasma levels of RA, observed in continuous RA therapy with a once-daily oral dose (110 $\frac{mg}{m^2}$) of RA, did not affect TM-mediated reduction of thrombin generation significantly. This finding prompts the hypothesis that continuous RA treatment has more consistent therapeutic effects on coagulation disorders than on cancer.

**Conclusions:**

Our results indicate that the oscillatory upregulation of TM expression on the endothelial cells over the course of RA therapy could potentially contribute to the treatment of coagulation abnormalities in cancer patients. Further studies on the impacts of RA therapy on the procoagulant activity of cancer cells are needed to better elucidate the mechanisms by which RA therapy improves hemostatic abnormalities in cancer.

**Electronic supplementary material:**

The online version of this article (10.1186/s12976-019-0099-z) contains supplementary material, which is available to authorized users.

## Background

All-trans retinoic acid (RA) plays key roles in cancer treatment and prevention. Breast, lung, bladder, prostate, and acute promyelocytic leukemia (APL) cancers were shown to be suppressed by RA [1–5]. RA therapy can also improve blood clotting disorders such as thrombosis and disseminated intravascular coagulation (DIC) in cancer patients [6–12]. DIC, a life-threatening coagulation disorder associated with uncontrolled clot formation and/or excessive bleeding, was reported in patients with different types of cancer [13–16]. Some of the mechanisms involved in the occurrence of DIC in cancer patients are known, and others are still under investigation. Tissue factor (TF) upregulation by tumor cells is one of the main causes of the observed hypercoagulable state in cancer patients [17–20]. TF binds to factor VIIa and forms a complex which activates factors X and IX. Activation of factor X leads to formation of the prothrombinase complex, which converts prothrombin to thrombin. Expression of the cancer procoagulant (CP), a specific enzyme that directly activates factor X, by tumor cells is another important mechanism for the initiation of the coagulation cascade in cancer [21, 22]. Tumor cells can also affect the coagulation cascade through interactions with other cell types such as monocytes and endothelial cells. Previous studies showed that circulating tumor cells increased the expression of TF by monocytes and endothelial cells [17, 23–25]. Platelet aggregation and induction of inflammatory cytokine release are the other phenomena which can be responsible for blood clotting system abnormalities in cancer patients [26, 27].

Clinical studies have indicated that RA treatment improved the plasma levels of hemostatic markers such as D-dimer, thrombin-antithrombin complex, and fibrinogen in APL patients in hypercoagulable states [11, 12]. Theories have been proposed to explain how RA therapy improves coagulation disorders. In vitro studies showed that RA significantly decreased the expression of TF in cancer cells [28, 29]. An in vivo study on the procoagulant activity of bone marrow blasts from APL patients under RA treatment revealed that TF and CP in the patients’ marrow blasts decreased after RA therapy [12]. RA can also affect the fibrinolytic system by increasing the synthesis of urokinase plasminogen activator (u-PA) in cancer cells and tissue plasminogen activator (t-PA) in endothelial cells [30, 31]. Thereafter, however, RA induces the expression of plasminogen activator inhibitors (PAIs), such as PAI-1 and PAI-2 [32]. The way these two contradictory pathways influence fibrinolysis in cancer patients has not been fully understood. RA also affects the procoagulant and anticoagulant properties of endothelial cells and monocytes [33, 34]. Previous studies have reported that RA increased the antithrombotic potential of microvascular endothelial cells by downregulating TF and upregulating thrombomodulin (TM) expression [34–36]. TM, a surface high-affinity receptor for thrombin, plays a key role in activation of the protein C (PC) anticoagulant pathway. Activated PC, produced by the TM-thrombin complex, inactivates cofactors Va and VIIIa, thus inhibiting thrombin generation. Although TM has significant effects on the blood coagulation system, its role in RA treatment of coagulation disorders in cancer patients has not yet been studied. Furthermore, to the best of our knowledge, there is no experimental or computational study that has investigated the extent and forms of variation in TM levels over the course of RA therapy in cancer. Thus, the main objective of this study was to investigate if, how and to what extent the RA-induced TM upregulation over the course of RA therapy with a single daily oral dose of RA affects thrombin generation profiles in cancer patients. Analysis of the variations of the thrombin generation profile is a classic/standard way of studying the significance of blood factors in the coagulation cascade. In this regard, we developed an ordinary differential equation (ODE) model of gene expression for the RA-induced upregulation of TM concentration on the endothelium. The expression rate of TM on the endothelium depends on the rate of RA diffusion from plasma into the endothelial cells. In plasma, a large fraction of RA (~ 99%) circulates bound to albumin. However, only free RA molecules in plasma can diffuse passively across the endothelial cell membrane and subsequently bind to RA receptors and activate transcription of the TM gene. The large amount of bound RA in plasma acts as a reservoir from which the RA is slowly released to the unbound form to maintain the equilibrium. Thus, we derived a new formula which expresses the TM transcription rate as a function of free RA concentration. Coupling the gene expression model with three other models, namely a two-compartment pharmacokinetic model of RA, an sTM release model and a mechanistic model of the human coagulation cascade, we investigated the effects of RA-induced TM upregulation on thrombin generation. Our results indicated that overexpression of TM over the course of RA therapy with a daily oral dose of 45 $\frac{mg}{m^2}$ or 110 $\frac{mg}{m^2}$ reduced thrombin level significantly. We also investigated how the progressive reduction in the plasma concentrations of RA over the course of continuous RA therapy with a single daily dose of (110 $\frac{mg}{m^2}$) RA can affect the corrective effects of RA therapy on thrombin generation. Increasing reductions in plasma concentration of RA over the course of RA treatment with a constant daily dose of RA is a potential sign of RA resistance at least in some cancer patients. The exact mechanism of development of resistance to RA has yet to be determined. Genetic mutations of retinoic acid receptors, increased metabolism of RA, and upregulation of cellular retinoic acid binding proteins which play important roles in the RA signaling pathway [37], have been proposed as possible reasons for RA resistance [38]. Our model predictions of RA resistance effects on the efficacy of RA therapy in treatment of coagulation abnormalities are applicable only to those cancer patients whose plasma levels of RA decrease over continuous treatment days.

## Method

**Fig. 1:**
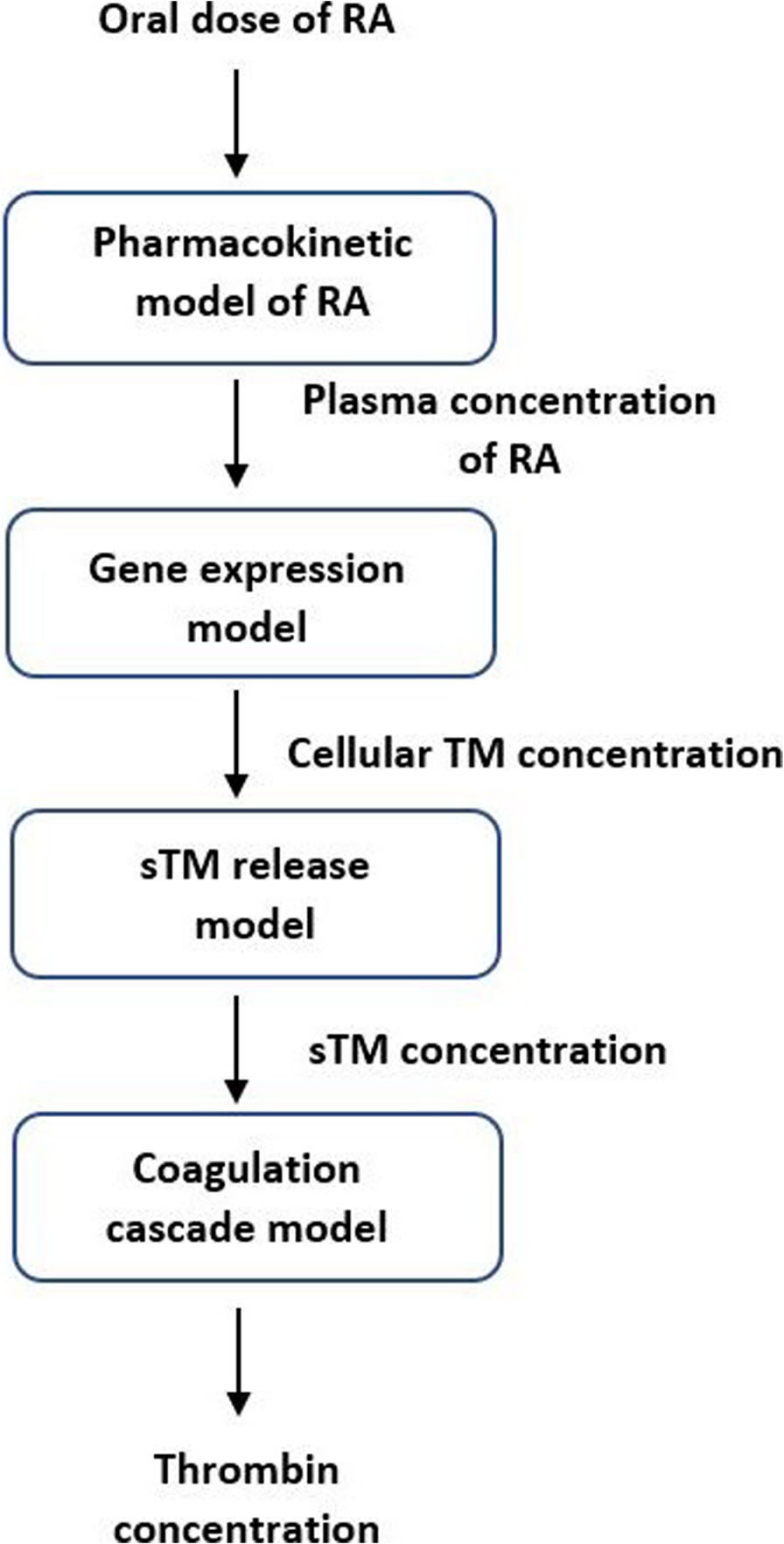
Coupling four models to study how taking an oral dose of RA affects thrombin generation. Each block represents a model, while the arrows before and after each block indicate the input and output of the model, respectively

In this section, we first develop a gene expression model that describes TM upregulation on the endothelial cell surface following RA treatment. We train the gene expression model using in vitro data from the literature. We then build a two-compartment pharmacokinetic model of RA, which describes the plasma concentration of RA in cancer patients. We couple the gene expression model with the pharmacokinetic model, to obtain the variations of TM level on the endothelial cell surface during the course of RA therapy. We then simulate the time-dependent variations of soluble thrombomodulin (sTM) concentration using an ODE model, called the sTM release model. Finally, we use the output of the sTM release model in an ODE model of the coagulation cascade to investigate the effects of RA-induced TM upregulation on thrombin generation. The gene expression model, pharmacokinetic model and sTM release model are explained in the following sub-sections, while the ODE model of the coagulation cascade is fully explained in [39]. Figure 1 shows the interactions between the different models in this study.

### Gene expression model

#### Experimental data

**Fig. 2:**
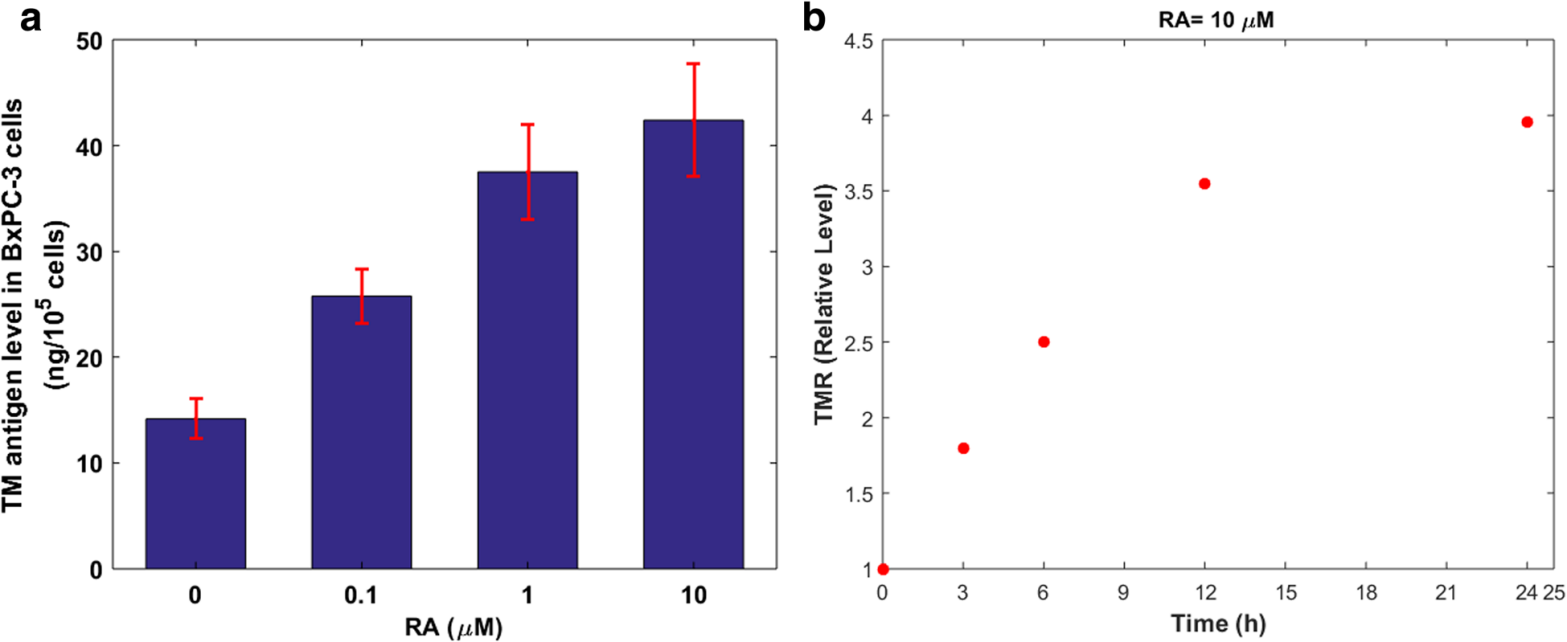
(a) Effects of RA treatment on TM levels in BxPC-3 cells. The reported values for TM level were obtained after treating the cells with RA for 24 h. Figure adopted from [40]. (b) Changes in relative levels of TMR in BxPC-3 cells treated with 10 μM RA. The mRNA level without incubation is defined as 1. Figure adopted from [40]

There are several lines of evidence regarding the upregulation of TM gene expression by RA [35, 40, 41]. This upregulation is due to transcriptional changes in the TMR expression level [41]. In this study, we used the experimental data presented by Horie et al. [40], which includes time-dependent variations in TMR levels, and dose-dependent changes in TM levels after treating human pancreas BxPC-3 cells with RA (Fig. 2). Human pancreas BxPC-3 cells were used in that study, as their characteristics of RA-dependent TM expression are the same as those of endothelial cells [40]. The cultured BxPC-3 cells became confluent with.

The cultured BxPC-3 cells became confluent with fetal calf serum, whose major component is albumin, prior to adding RA to the medium. The TM levels in Fig. 2a were measured after treating the cells with various concentrations of RA for 24 h. The relative values for TMR level in Fig. 2b were obtained after treating the cells with 10 μM of RA.

#### Formulation of the model

We formulated an ODE model to study the RA-induced upregulation of TM gene expression. The model included gene transcription, protein translation, and mRNA and protein degradation. The model consisted of two species, namely TM and TMR, with ten parameters (Table 1).

1 $$ \frac{\mathrm{d}\left[\mathrm{TMR}\right]}{\mathrm{d}\mathrm{t}}=I+{I}_0-{k}_{\mathrm{d}\mathrm{m}}\left[\mathrm{TMR}\right] $$

2 $$ \frac{\mathrm{d}\left[\mathrm{TM}\right]}{\mathrm{d}\mathrm{t}}={k}_{\mathrm{trans}}\left[\mathrm{TM}\mathrm{R}\right]-{k}_{\mathrm{d}\mathrm{p}}\left[\mathrm{TM}\right], $$

**Table 1:** List of the model parameters

Parameters	Description	Range	Reference
*I*_0_	Basal transcription rate by transcription factors, which are not affected by RA (M/h)	1.6 × 10^−12^ − 1.6 × 10^−9^	[73]
*k*_dm_	TMR degradation rate (1/h)	0.256	[71]
*k*_trans_	Translation rate(1/h)	1–10,000	[73]
*k*_dp_	TM degradation rate (1/h)	0.0845	[72]
*IC*_TMR_	TMR initial concentration (M)	1.6 × 10^−11^ − 1.6 × 10^−8^	[73, 74]
*IC*_TM_	TM initial concentration (M)	1.65 × 10^−5^ − 2.14 × 10^−5^	[40]
*I*_max_	Maximal transcription rate of the TM gene (M/h)	1.6 × 10^−12^ − 1.6 × 10^−9^	[73]
*k*_d1_	Equilibrium dissociation constant of RA binding to retinoic acid receptor (M)	8 × 10^−9^	[75]
*k*_d2_	Equilibrium dissociation constant of DNA-transcription factor complex (M)	15 × 10^−9^	[76]
[REC_1t_]	Total concentration (M) of the specific transcription factor which can activate the TM gene transcription	1 × 10^−11^ − 1 × 10^−6^	Unknown. A large range is used.

where [TMR] and [TM] indicate molar concentration of TMR and TM, respectively. [TM] and [TMR] are functions of time. The molar concentration of TM, which is a membrane-bound protein, was calculated by dividing the number of moles of TM by the cell volume. The cell volume was set to [42–44].

$$ {V}_{\mathrm{cell}}={10}^{-13}\mathrm{L}. $$

The transcription rate (*I*) of the TM gene was the only parameter in the model that depended on RA concentration. An increase in RA concentration leads to activation of a transcription factor, which is responsible for the activation of the TM gene. Considering the mechanism of action of RA, we derived an expression for the TM transcription rate (*I*), (Proof in Additional file 1)

3 $$ I={I}_{\mathrm{max}}\frac{\left[\mathrm{RA}\right]\left[{\mathrm{REC}}_{1\mathrm{t}}\right]}{\left[\mathrm{RA}\right]\left[{\mathrm{REC}}_{1\mathrm{t}}\right]+{k}_{\mathrm{d}2}\left(\left[\mathrm{RA}\right]+{k}_{\mathrm{d}1}\right)}, $$

where [RA] and [REC_1t_] in Eq. 3 indicate free RA concentration and total concentration of the specific transcription factor which can activate the transcription of the TM gene, respectively. *I* is time-dependent, since [RA] can change over time, while *I*_max_ and [REC_1t_] are constant for the TM gene in a given cell type. The rest of the parameters in Eq. 3 are defined in Table 1. RA is highly bound to albumin in the culture medium [40] and in plasma [45]. We assumed that the unbound fraction of RA is about 1% of the total RA concentration [45, 46]. It is important to note that only the free drug in the culture medium or plasma is able to have a therapeutic effect.

The translation rate (*k*_trans_) and basal transcription rate (*I*_0_) were the only parameters that depended on the other parameters. Assuming that the TM and TMR concentrations were in steady state before RA treatment, we calculated *k*_trans_ and *I*_0_ by

4 $$ {k}_{\mathrm{trans}}=\frac{k_{\mathrm{dp}}{IC}_{\mathrm{TM}}}{IC_{\mathrm{TM}\mathrm{R}}}, $$

5 $$ {I}_0={k}_{\mathrm{dm}}{IC}_{\mathrm{TMR}}. $$

**Fig. 3:**
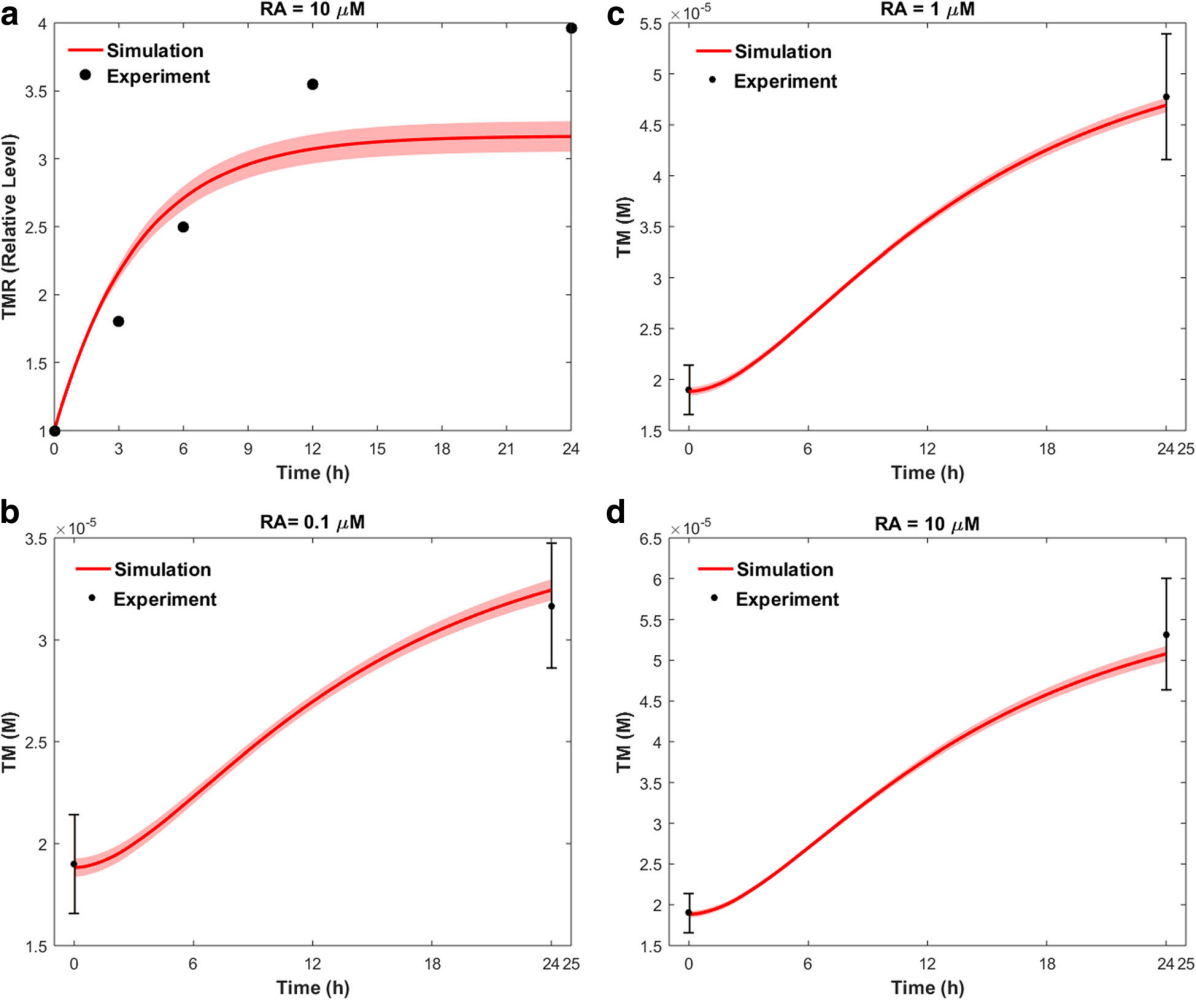
The gene expression model training simulations. The model’s unknown parameters were estimated using PSO. The red lines show the mean simulated results for (a) TMR, (b) TM at RA = 0.1 μM, (c) TM at RA = 1 μM and (d) TM at RA = 10 μM. The shaded regions denote 99% confidence interval of the mean results [47, 70]. The black dots indicate the experimental data, while the standard deviation of each experimental data point is half the length of the total error bar

The model had six unknown parameters, for which we considered some bounds (Table 1). The bounds for *IC*_TM_ were due to the experimental errors, while the other unknown parameters, namely *I*_max_, *IC*_TMR_, *k*_trans_, *I*_0_ and [REC_1t_] had physiological bounds. We estimated the model’s unknown parameters by minimizing the residual between simulation results and empirical measurements, following a parameter estimation algorithm. In this regard, we used a particle swarm optimization (PSO) technique [47] (See Additional file 1 for full details regarding the parameter estimation algorithm and PSO). Our simulation results for the time-dependent variations in TM and TMR concentrations compared reasonably well with the experimental data (Fig. 3).

The solid lines in Fig. 3 show the mean simulated results, while the shaded regions show the 99% confidence interval of the mean simulated results. From Fig. 3a, it can be seen that TMR reached steady state almost 18 h after administration of RA, while TM did not reach steady state even after 24 h (Fig. 3b-d).

**Fig. 4:**
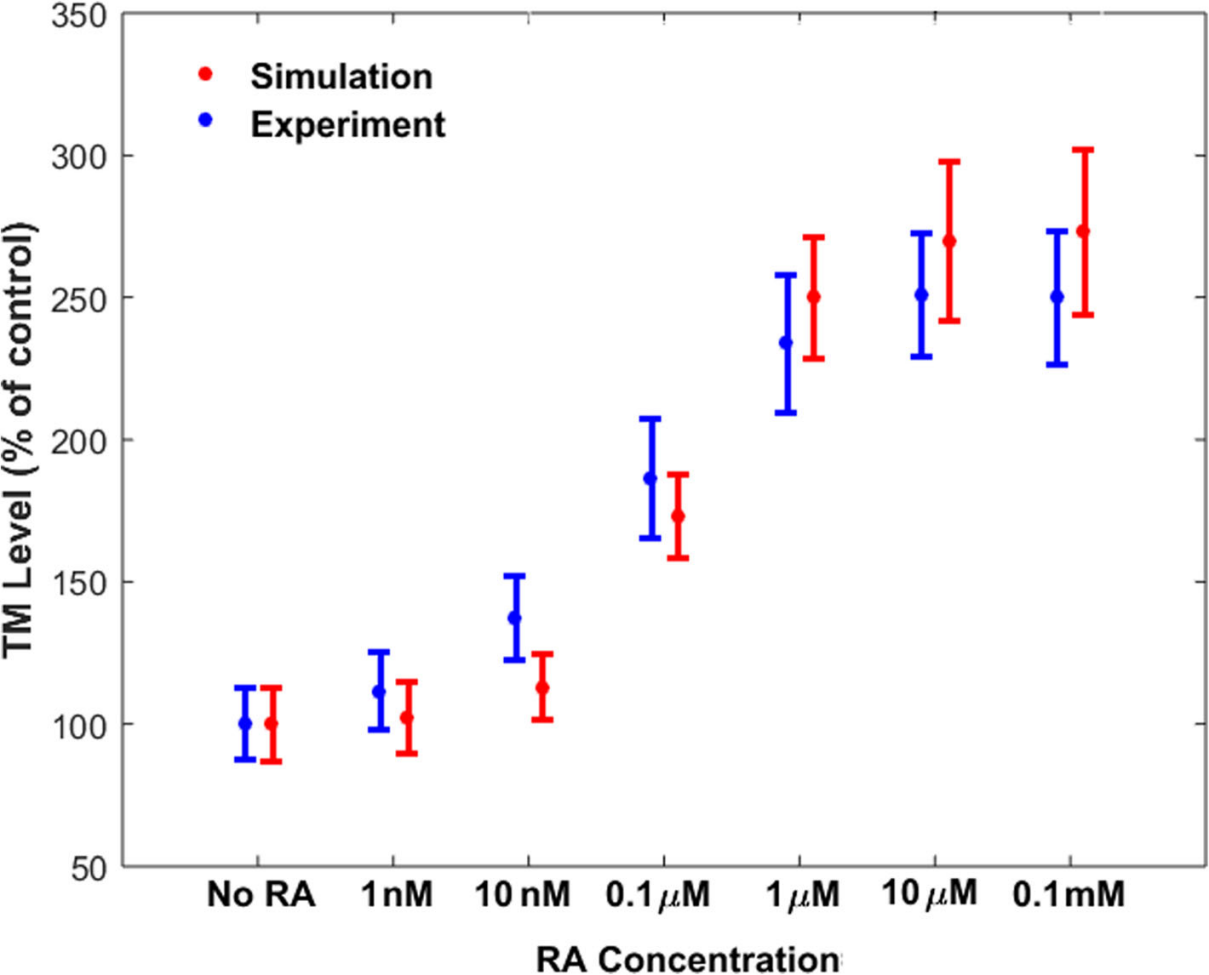
Comparison between the simulated results for fold change values of TM concentration after 24 h of treatment with various concentrations of RA, with experimental data not used during model training. The simulation results were obtained using the gene expression model trained by the experimental data shown in Fig. 2, while the experimental data in Figure was reproduced from [41]

Using the estimated parameters from the training data set [40], we compared the model’s predictions with another data set [41] for the RA-induced upregulation of TM on endothelial cells. In this regard, fold change values of TM concentration after 24 h of treatment with various concentrations of RA were obtained (Fig. 4). Figure 4 indicates that simulation results compared reasonably well with experimental data.

### Pharmacokinetic model

**Fig. 5:**
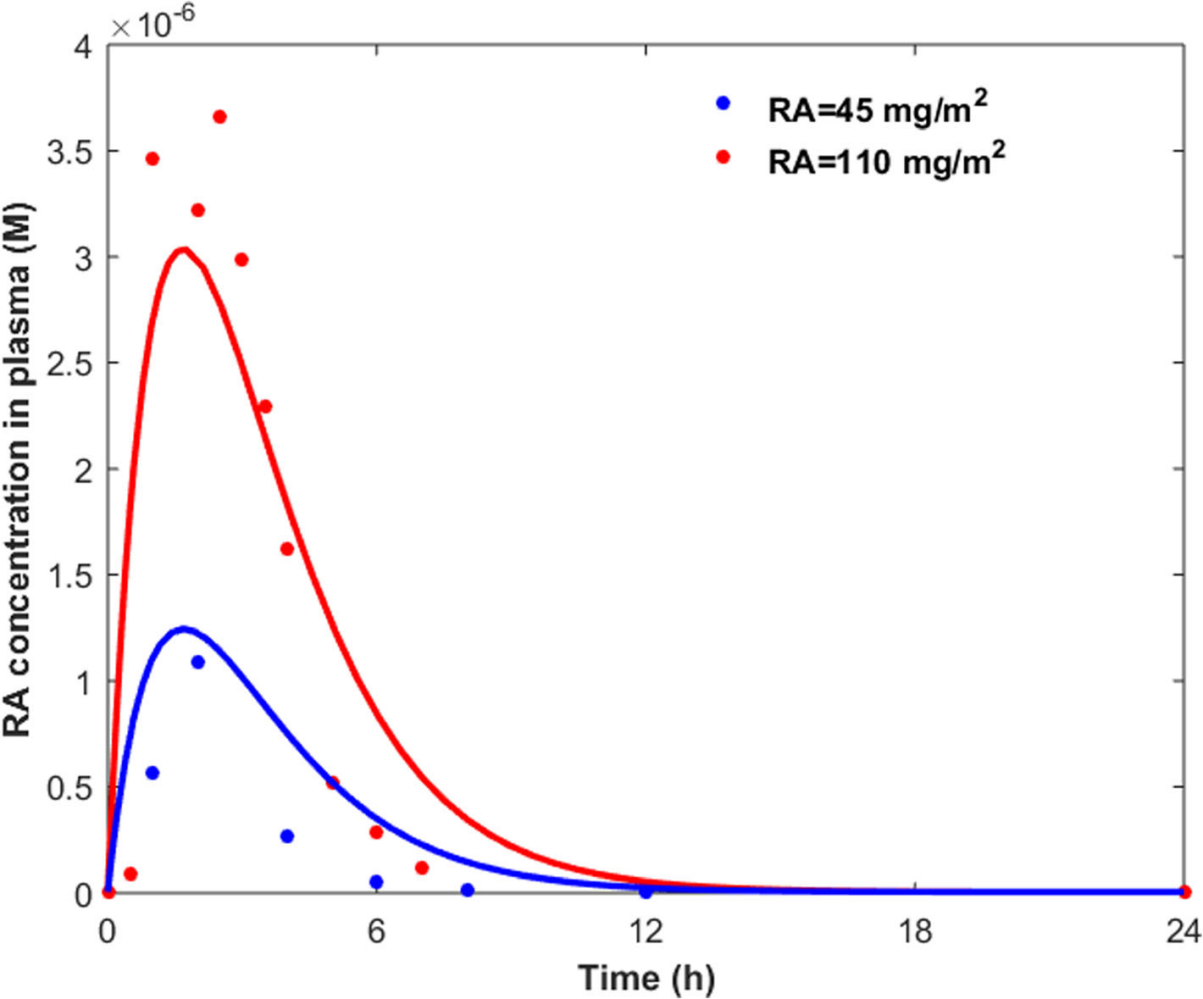
Plasma concentration of RA after ingestion of various doses of RA on day 1 of treatment [50, 57]. Dots show the pharmacokinetic data, while lines indicate the simulation results by a two-compartment pharmacokinetic model

Some cancer patients take RA as a part of their cancer treatment within 3 to 4 months of diagnosis [48]. The plasma concentration of RA changes significantly after oral administration of various doses of the drug (Fig. 5).

**Fig. 6:**
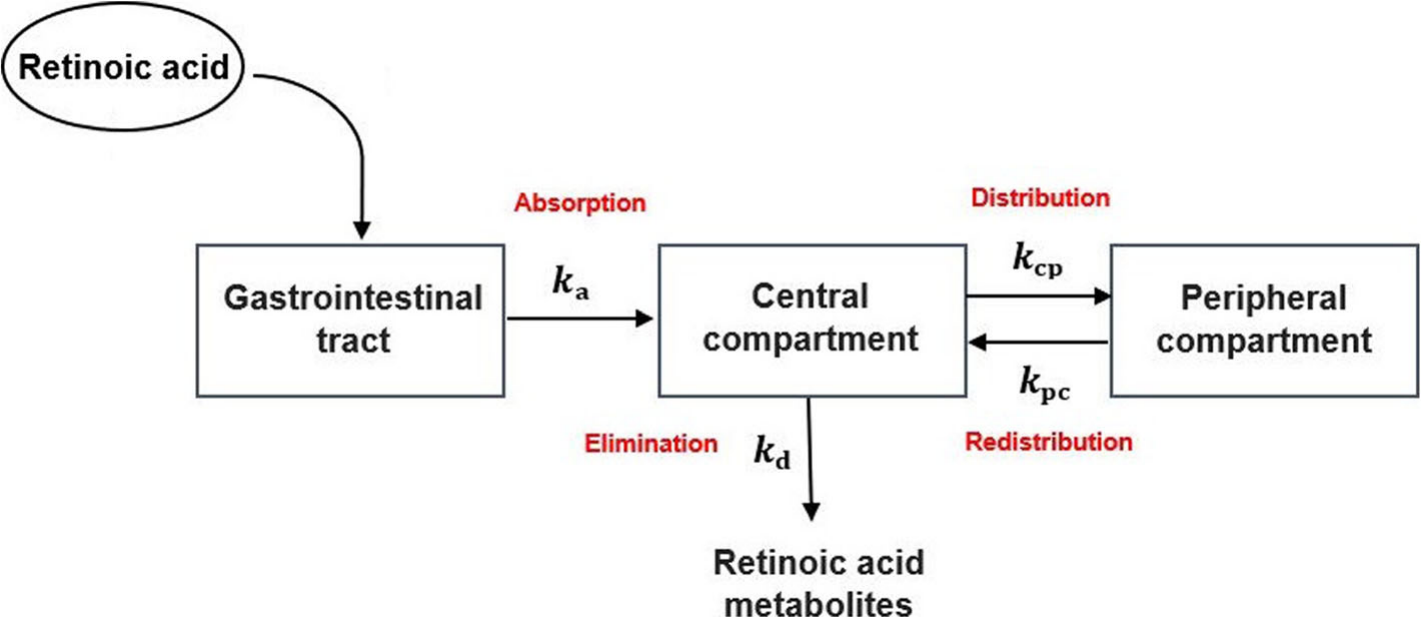
Schematic of a two compartments model for describing RA pharmacokinetics. The model included two main compartments namely, central compartment and peripheral compartment. *k*_a_, *k*_d_, *k*_cp_, *k*_pc_ are first order absorption rate constant, degradation rate constant, distribution rate constant and redistribution rate constant

Oral administration of RA can be modeled via a two-compartment pharmacokinetic model (Fig. 6), since the plasma concentration-time curve of RA exhibits a biexponential decline [49, 50]. After oral administration of the drug, RA is absorbed into the bloodstream, which is a part of the central compartment. The central compartment includes the plasma and organs, where the distribution of RA is assumed to be instantaneous. The RA is eliminated by a first order process from the central compartment or distributed to the rest of the body that represents the peripheral compartment. RA elimination mainly occurs in the liver and kidney, which are included in the central compartment. The peripheral compartment includes tissues where RA distribution occurs with a slower rate than in the central compartment.

RA exchange between different compartments can be described by a system of ODEs

6 $$ \frac{\mathrm{dD}}{\mathrm{dt}}=-{k}_{\mathrm{a}}\mathrm{D} $$

7 $$ \frac{\mathrm{d}\left[{\mathrm{RA}}_c\right]}{\mathrm{d}\mathrm{t}}=\frac{k_{\mathrm{a}}\mathrm{D}}{V_{\mathrm{c}}\mathrm{M}}-\left({k}_{\mathrm{d}}+{k}_{\mathrm{c}\mathrm{p}}\right)\left[{\mathrm{RA}}_{\mathrm{c}}\right]+\frac{k_{\mathrm{p}\mathrm{c}}{V}_{\mathrm{p}}}{V_{\mathrm{c}}}\left[{\mathrm{RA}}_{\mathrm{p}}\right] $$

8 $$ \frac{\mathrm{d}\left[{\mathrm{RA}}_{\mathrm{p}}\right]}{\mathrm{d}\mathrm{t}}=\frac{k_{\mathrm{c}\mathrm{p}}{V}_{\mathrm{c}}}{V_{\mathrm{p}}}\left[{\mathrm{RA}}_{\mathrm{c}}\right]-{k}_{\mathrm{p}\mathrm{c}}\left[{\mathrm{RA}}_{\mathrm{p}}\right] $$

where D, [RA_c_] and [RA_p_] represent RA dose, total RA concentration in the central compartment, and total RA concentration in the peripheral compartment, respectively. D, [RA_c_] and [RA_p_] vary over time, and are represented in g, molar and molar, respectively. *k*_a_, *k*_d_, *k*_cp_, *k*_pc_, *V*_c_ and *V*_p_ are the first-order absorption rate constant, first-order degradation rate constant, distribution rate constant, redistribution rate constant, central compartment volume and peripheral compartment volume, respectively. M is the molar mass of RA, and is set to 300.4 gmol^− 1^. We also assumed that the average body surface area is 1.75 m^2^. We built the model, shown in Fig. 6, in MATLAB SimBiology and fit the model parameters to the pharmacokinetic data (dots in Fig. 5). Solid lines in Fig. 5 show the model prediction for plasma concentration of RA ([RA_c_]) after taking an oral dose of 45 or 110 mg/m^2^ of RA. Further details regarding the pharmacokinetic model were provided in the Additional file 1. We coupled the gene expression model with the pharmacokinetic model to study the effects of oral administration of RA on the expression levels of TM and TMR during the course of RA therapy (Pharmacokinetics-pharmacodynamics). Free RA concentration in the gene expression model ([RA] in Eq. 3) is obtained by

9 $$ \left[\mathrm{RA}\right]=0.01\left[{\mathrm{RA}}_{\mathrm{c}}\right]. $$

### sTM release model

TM plays a key role in controlling fibrin formation. A modified form of TM is also found in human plasma and urine [51]. Both cellular TM and soluble TM (sTM) act as an anticoagulant by activating protein C [52, 53]. It is believed that soluble TM (sTM) is a marker for endothelial cell injury [54, 55]. Endothelial cell injury can occur due to several reasons, such as elevated levels of cytokines, hyperlipidemia, activation of leukocytes and neutrophils, hypercholesterolemia, obesity, diabetes and smoking. Blood vessel damage is also a common occurrence in cancer patients, as many of them undergo surgery or chemotherapy. It has been reported that TM is cleaved from the endothelium and released into the plasma by some degrading enzymes such as protease and glycosidase upon endothelial cell injury [54]. Assuming the RA therapy does not affect the mechanisms mediating the release of cellular TM into the plasma, we modeled the rate of sTM production by

10 $$ \frac{\mathrm{d}\left[\mathrm{sTM}\right]}{\mathrm{d}\mathrm{t}}={c}_1\left[\mathrm{TM}\right]-{c}_2\left[\mathrm{sTM}\right], $$

where *c*_1_ and *c*_2_ are the release rate constant of cellular TM into the plasma by the degrading enzymes and the elimination rate constant of sTM from plasma, respectively. [TM] represents the cellular concentration of TM, while [sTM] indicates the plasma concentration of sTM. *c*_2_ was set at 0.11 $\frac{1}{h}$ [56].

Assuming the sTM concentration to be at steady state prior to RA treatment, we calculated the value of *c*_1_ using the physiological concentrations of TM and sTM

11 $$ {c}_1=\frac{c_2{\left[\mathrm{sTM}\right]}_0\ }{{\left[\mathrm{TM}\right]}_0}, $$

where [sTM]_0_ and [TM]_0_ are physiological concentrations of sTM and TM, and are expressed in molar. We assumed that [sTM]_0_ = 1nM, while [TM]_0_ is the steady state level of TM in Eq. 2 after treating the model with a physiological concentration of RA (RA_plasma_ = 5 nM) [46].

The sTM release model was used to link the gene expression model to the ODE model of the coagulation cascade.

## Results

### Pharmacokinetics-pharmacodynamics

RA is mainly transported in plasma bound to serum albumin [45, 50]. The unbound fraction of RA in plasma is about 1% of the total RA concentration [45, 46]. Since the bound drugs are pharmacologically inactive, we calculated the unbound fraction of RA in plasma by multiplying the total concentration of RA by 0.01 (Eq. 9). We then used the plasma concentration of free RA as input to the gene expression model, with the parameters estimated in the Gene expression model, to study the dynamics of the TM concentration on the first day of treatment. To do so, the free RA concentration in Eq. 3 varied according to the time-dependent levels of free drug in plasma ([RA]) following RA therapy.

**Fig. 7:**
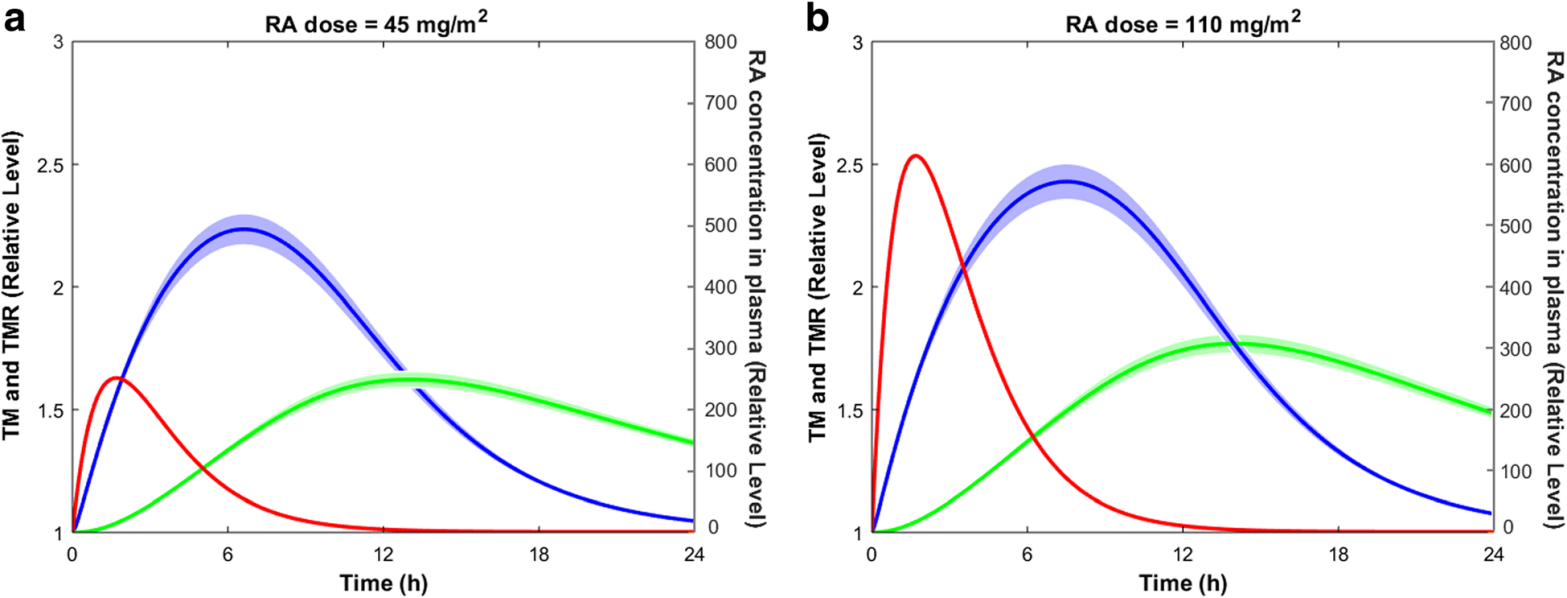
Variations in RA, TMR, and TM concentrations following a single dose of RA (a) $45\frac{mg}{m^2}$ and (b) 110 $\frac{mg}{m^2}$ . The lines show the mean simulated results, while the shaded regions denote the 99% confidence interval of the mean simulated results

RA treatment resulted in variations in TM and TMR concentrations (Fig. 7). The TMR and TM concentrations reached their peak levels almost 7 and 13 h after taking a 45 mg/m^2^ oral dose of RA, respectively (Fig. 7a). However, the peak times of TMR and TM levels were shifted by almost 1 h when the RA dose was increased to 110 mg/m^2^ (Fig. 7b).

**Fig. 8:**
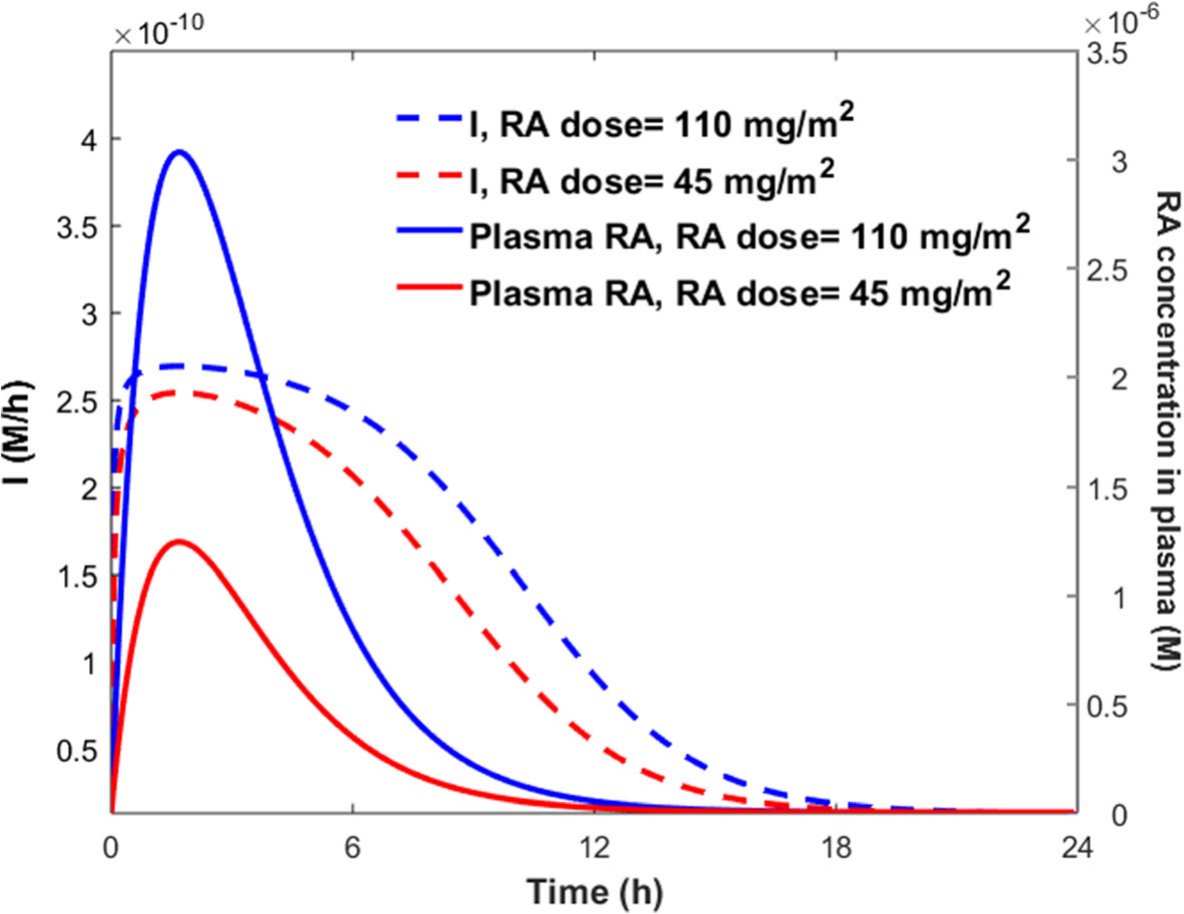
Variations of the TM transcription rate (*I*), and plasma concentration of RA following various doses of RA

The solid lines in Fig. 7 denote mean simulated results, while the shaded regions denote 99% confidence interval of the mean simulated values. The maximum concentration of TM after taking a 110 $\frac{mg}{m^2}$ oral dose of RA (Fig. 7b) was similar to that of taking a 45 $\frac{mg}{m^2}$ oral dose of RA (Fig. 7a). This is because the transcription rate levels were comparable for both RA doses (Fig. 8).

### Continuous treatment with RA

**Fig. 9:**
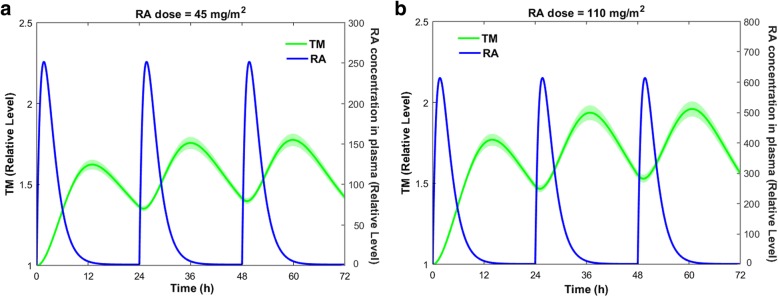
TM expression on the endothelial cell surface within three days of RA treatment. Daily doses of RA are taken at t = 0 h, t = 24 h, and t = 48 h. (a) $45\frac{mg}{m^2}$ and (b) 110 $\frac{mg}{m^2}$. The lines show the mean simulated results, while the shaded regions denote the 99% confidence interval of the mean simulated results

Patients on RA therapy usually take the drug on a daily basis. We simulated the effects of daily administration of RA for 3 days, on TM expression (Fig. 9).

Continuous treatment with RA resulted in oscillatory alterations in the TM concentration. These oscillatory changes are important, as they can affect the blood coagulation cascade. Taking 110 $\frac{mg}{m^2}$ RA per day increased the TM concentration to approximately twice its normal level (i.e. no RA treatment) almost 14 h after drug ingestion. Daily administration of RA did not allow the TM level to return to its initial concentration, since it took almost 72 h for TM to return to its initial concentration (data not shown).

#### RA resistance

**Fig. 10:**
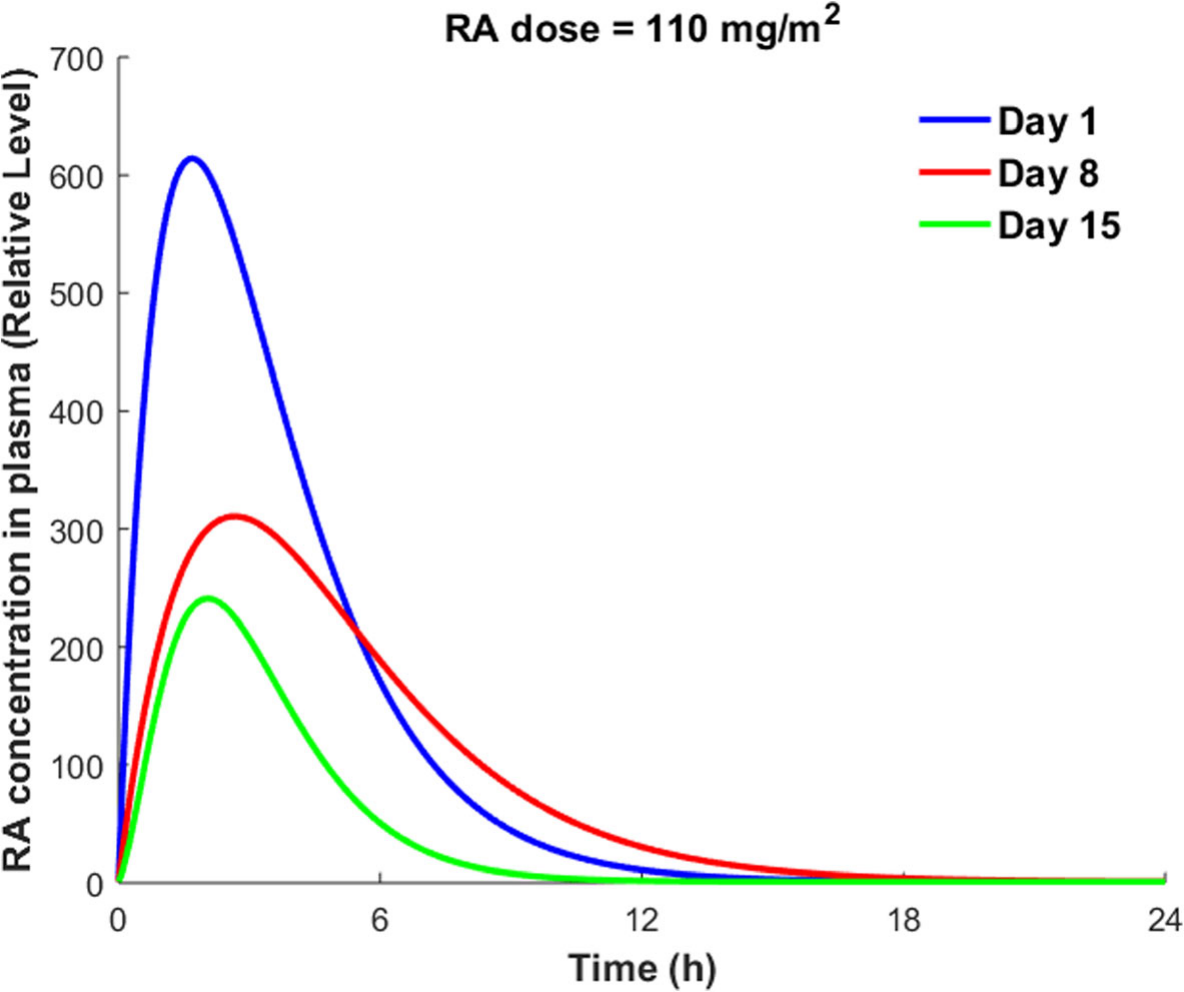
Changes in the total plasma level of RA concentration on treatment days 1, 8 and 15 of a continuous treatment period with daily dose of 110 $\frac{mg}{m^2}$ RA [57]

In some cancers, RA resistance is associated with increasing reductions in the plasma concentration of RA [50]. A clinical trial of RA [57] showed that continuous treatment with RA caused a progressive reduction in the plasma level of RA in half of the patients that were on RA treatment (Fig. 10). The mechanisms involved in the progressive reduction in RA plasma concentration over the course of continuous RA therapy are not known. The mechanisms might be cancer- and patient- specific. Other pharmacokinetic patterns were observed in the remainder of the patients under study [57]. In some patients, the peak plasma level of RA remained unchanged during the RA treatment, while other patients had peaks that varied weekly.

**Fig. 11:**
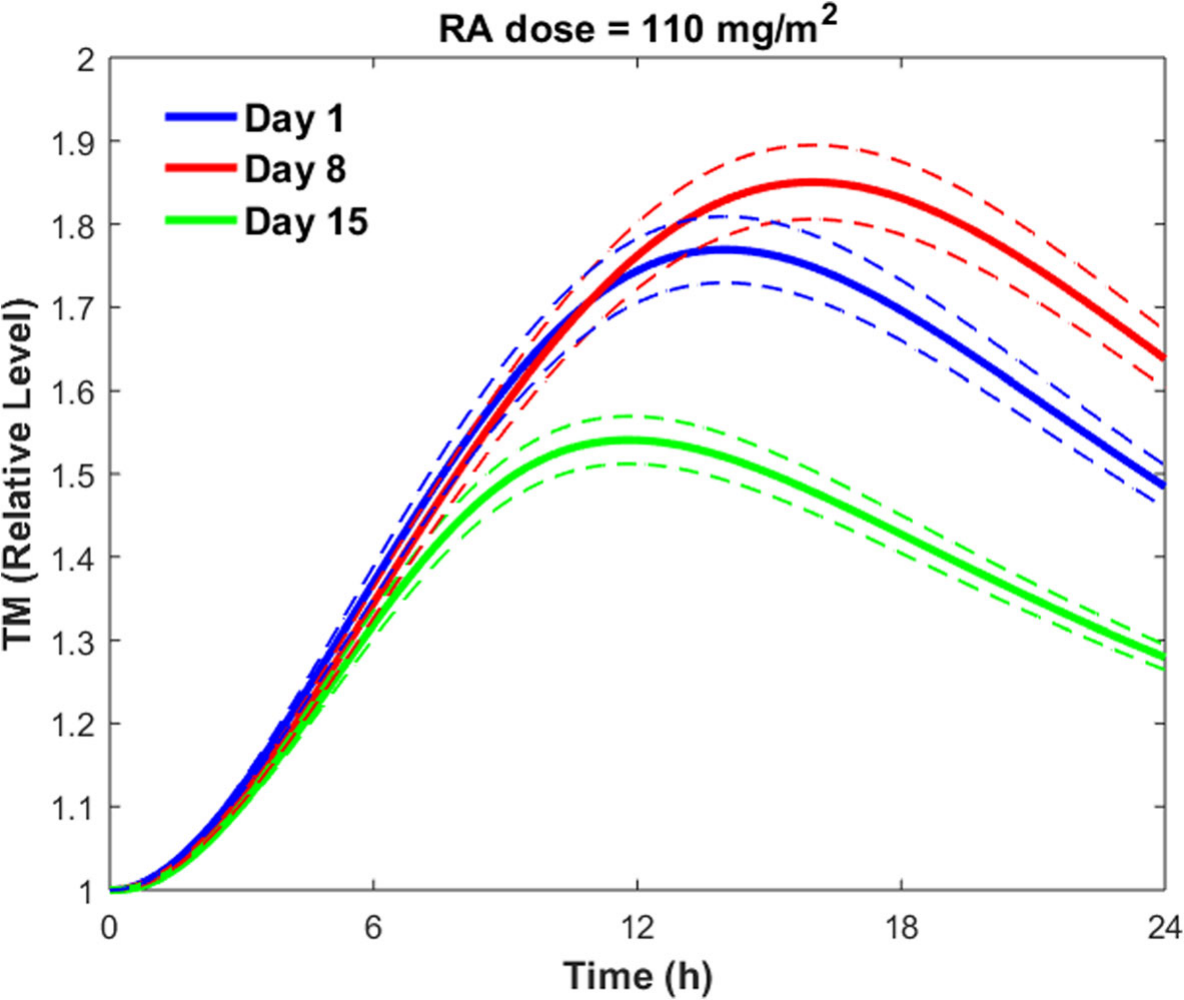
TM expression on day 1, day 8, and day 15 of the treatment period. Solid lines are the mean simulated values. Dotted lines show the 99% confidence estimate of the mean results

Using the clinical data shown in Fig. 10, we simulated the effects on TM expression of the consistent decrease in peak plasma level of RA (Fig. 11).

The solid lines in Fig. 11 show the mean simulated values of the TM concentration, while the dotted lines denote the 99% confidence interval of the mean results. Figure 11 shows that the peak level of TM on various days decreased in the order of day 8 > day 1 > day 15, while the peak RA plasma concentration decreased in the order of day 1 > day 8 > day 15. Higher TM levels on day 8 compared to day 1 was because of higher plasma levels of RA after 6 h of drug administration on day 8 compared to day 1 (Fig. 10). The results presented in Fig. 11 were obtained using the pharmacokinetic data from [57]. Thus, these results are not applicable to all patients with different cancer types. However, the current model can be used to study the variation of TM expression over the course of RA therapy for different patients with different cancer types, once more pharmacokinetic data on different treatment days is available. In the next section we will investigate the effects of RA-induced TM upregulation on the coagulation cascade. We also investigate how the progressive reduction in the RA concentration over the course of RA therapy can decrease the corrective effects of RA therapy on the coagulation disorders.

### Effects of RA-induced TM upregulation on the blood coagulation system

#### Effects of continuous RA therapy on thrombin generation

**Fig. 12:**
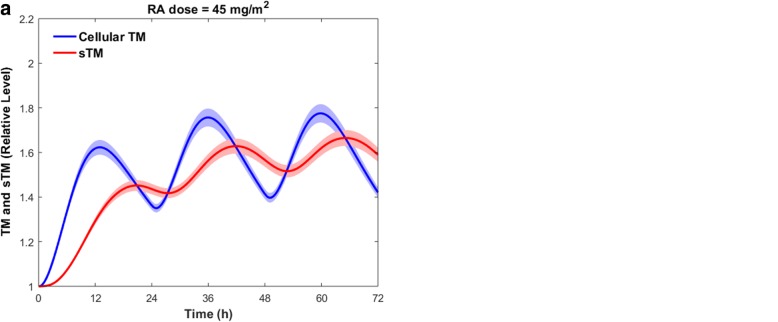
Variations in the cellular level of TM and the plasma concentration of sTM within three days of RA treatment. Daily doses of RA are taken at t = 0 h, t = 24 h, and t = 48 h. (a) $45\frac{mg}{m^2}$ and (b) 110 $\frac{mg}{m^2}$. Solid lines are the mean simulated values. Shaded regions show the 99% confidence interval of the mean results

In this section, we investigate whether the elevated levels of TM over the course of RA therapy can affect thrombin generation. In this regard, we used an ODE model of the blood coagulation cascade that incorporates a mechanistic description of the protein C pathway [39]. The coagulation model was developed based on in vitro phospholipid-based assays to study the contribution of various coagulation factors to thrombin generation in protein C deficient patients. TM was modeled at 1 nM in that study, which is an estimate of the physiological concentration of soluble TM (sTM) in plasma. However, our gene expression model predicts the variation in the concentration of TM in endothelial cells. It is believed that sTM is entirely derived from the TM expressed on the endothelial cell surface. To couple the RA model with the coagulation model, we need to obtain the time-dependent variations in the sTM level following the RA therapy. In this regard, we coupled the gene expression model with the sTM release model to obtain the variations of sTM over the course of RA therapy. Figure 12 compares the variation of the cellular level of TM with that of the plasma level of sTM when the patient takes the drug on a daily basis for three consecutive days.

**Fig. 13:**
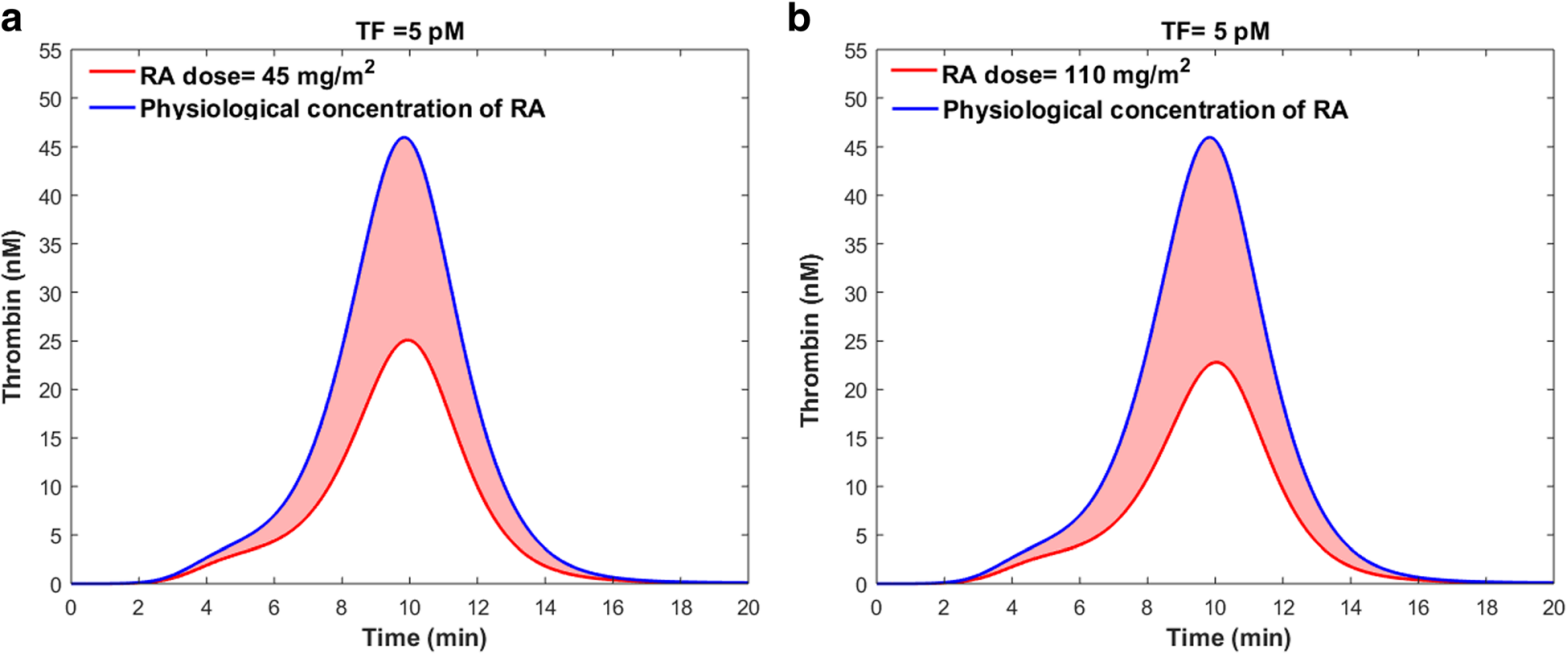
Impacts of RA-induced TM upregulation following drug ingestion (a) $45\frac{mg}{m^2}$ and (b) 110 $\frac{mg}{m^2}$, on thrombin generation. The blue lines indicate the thrombin generation profile in the control group with physiological levels of RA. The red lines show the maximum impact of RA therapy on thrombin generation. The shaded red regions indicate the range of the thrombin generation profile within three days of continuous RA therapy

To run the coagulation model, we assumed that all coagulation factor concentrations were physiological concentrations, except the TF and sTM concentrations. The TF concentration was set to 5 pM to initiate the clot formation process, while the sTM concentration varied according to the RA treatment (Fig. 12). Since the time scale of the coagulation cascade (20 min) is much shorter than that of TM expression (days), the sTM concentration was assumed to be constant during the coagulation process. Taking once-daily oral dose 45 $\frac{mg}{m^2}$ or 110 $\frac{mg}{m^2}$ of RA for three consecutive days reduced the peak level of thrombin up to 45 and 50%, respectively (Fig. 13). The endogenous thrombin profile (ETP), which is defined as the time integral of thrombin generation, was decreased up to 45 and 49% within 3 days of treatment with 45 $\frac{mg}{m^2}$ or 110 $\frac{mg}{m^2}$ oral dose of RA, respectively (Fig. 13).

The shaded regions in Fig. 13 indicate the range of thrombin generation after RA therapy, with different doses of RA. The shaded regions in Fig. 13 are not only due to the uncertainty of the results caused by the error bars in the experimental data (Fig. 2), but also due to the changes in sTM level after RA treatment (Fig. 12). In fact, we obtained the range of thrombin generation profiles using different values of sTM, that could be expected over the course of RA therapy (Fig. 12). Our results indicate that the endothelium could potentially play a key role in RA treatment of coagulation disorders, by upregulating TM and sTM. Since the sTM concentration fluctuates over time, the efficacy of RA treatment in preventing or treating hemostatic abnormalities is dependent upon the timing of the treatment.

#### Effects of RA resistance on thrombin generation

**Fig. 14:**
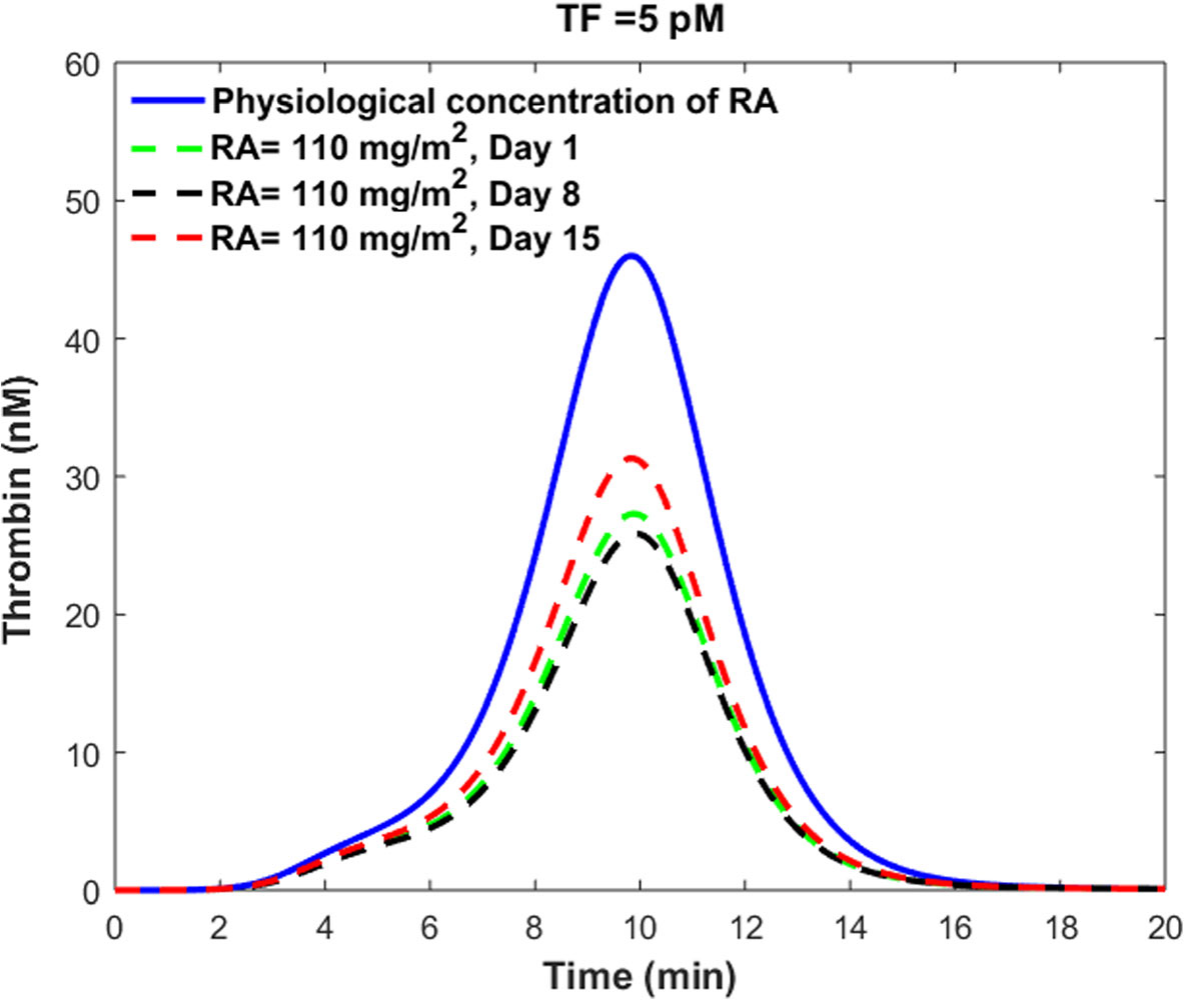
Thrombin generation profile on days 1, 8 and 15 of treatment period with a daily dose of 110 $\frac{mg}{m^2}$ RA. Dashed lines show the maximum impact of RA therapy on thrombin generation on different treatment days. The area between each dashed line and the solid blue line indicates the range of the thrombin generation profile on different treatment days. RA concentration in plasma on different treatment days is shown in Fig. 10

Our results indicated that the progressive reductions in plasma concentration of RA over the course of RA therapy with a daily oral dose (110 $\frac{mg}{m^2}$) of RA (Fig. 10) resulted in variations of cellular TM concentration (Fig. 11). Using the sTM release model, we obtained the variations of sTM concentration on different treatment days. We then ran the coagulation model using the predicted values of sTM concentration on different treatment days (Fig. 14), according to the procedure explained in the previous section. The blue line in Fig. 14 indicates the thrombin generation profile for the control condition with a physiological level of RA, while the dashed lines show thrombin generation profiles at sTM peak time after taking different oral doses of RA. Thus, the area between the blue line and a given day’s dashed line shows the range of variation of thrombin generation on the given day.

Our simulation results indicated that the peak thrombin level was reduced up to 41, 44 and 32% on day 1, day 8 and day 15 of treatment period, respectively. However, the ETP was reduced up to 40, 43 and 32% on days 1, 8 and 15 of continuous treatment with a once-daily dose of 110 $\frac{mg}{m^2}$ RA, respectively. It is important to note that the maximum reduction happens at the sTM peak time after oral administration of RA.

Our results indicated that the reduced levels of RA on days 8 and 15 of continuous treatment with a single daily dose of 110 $\frac{mg}{m^2}$ RA could decrease thrombin peak levels and ETP significantly. However, the way this progressive reduction affects the efficacy of RA in treating cancer depends on cancer type, stage and the patient’s health conditions. In general, drug dose and route of administration are determined in such a way that the plasma concentration of drug lies within the therapeutic window of the drug. Any significant reduction in plasma concentration of RA over the course of treatment can potentially decrease the efficacy of RA in treatment of cancer in at least some patients. Figure 10 indicates that the peak plasma concentration of RA decreases by almost 60% within two weeks of RA treatment, while our results show that the peak thrombin level is reduced up to 44 and 32% on days 8 and 15 of treatment, respectively. The obtained values for percent decrease in peak thrombin level on days 8 and 15 of RA therapy are comparable with a 41% decrease in peak thrombin level on day 1 of treatment. Our results raise the hypothesis that RA therapy has more consistent, corrective effects on clotting abnormalities than on cancer. Further studies on different patients with different cancer types and stages are needed to reveal how the observed reductions in plasma levels of RA over the course of RA therapy affect the efficacy of RA in treatment of cancers versus hemostatic abnormalities.

#### Effects of physiological levels of RA on thrombin generation

**Fig. 15:**
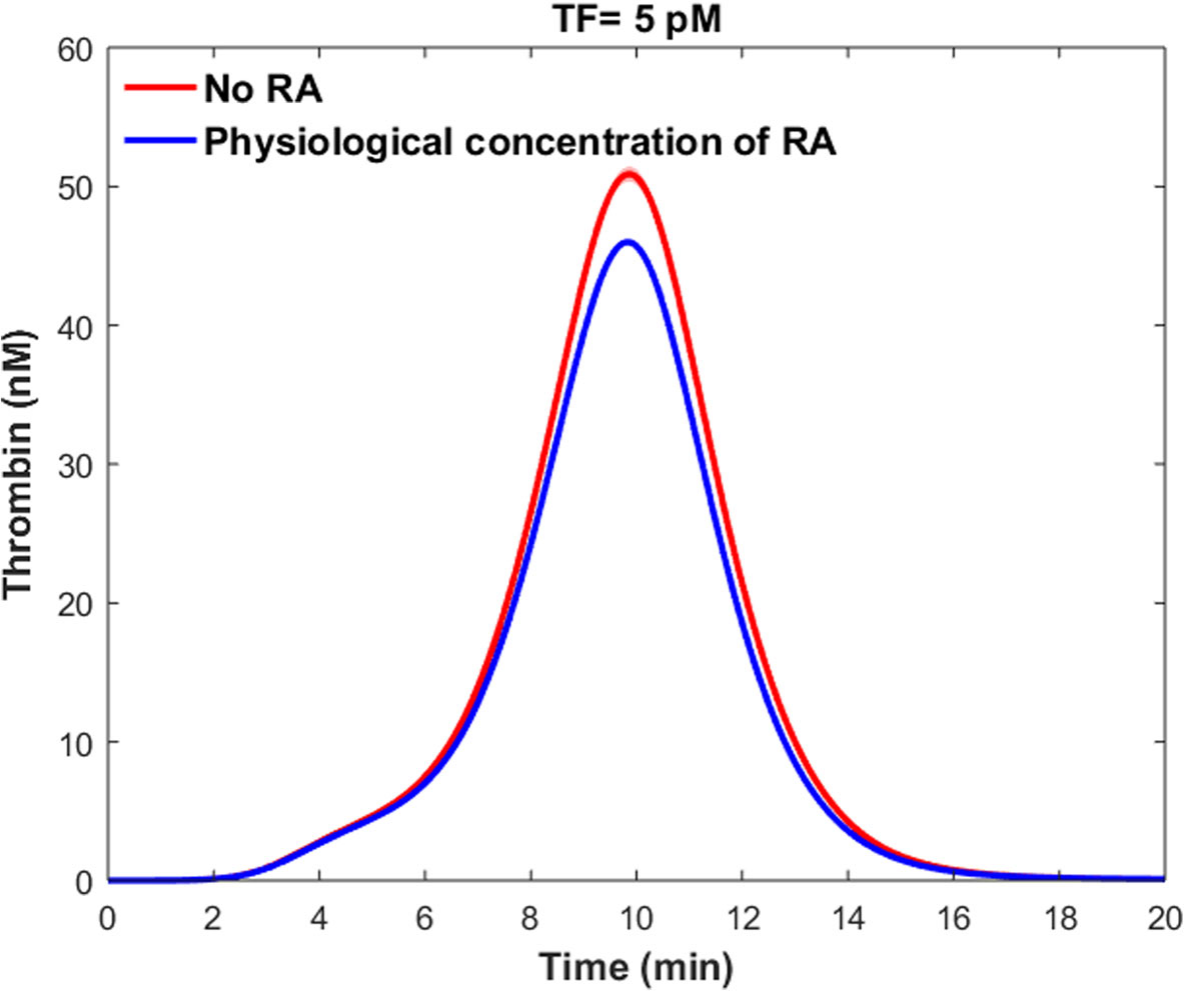
Impacts of physiological levels of RA on thrombin generation profile. The blue line indicates thrombin generation profile for the control condition with physiological level of RA. The red line shows the mean thrombin generation profile when there is no RA in the plasma. The shaded red region indicates the 99% confidence interval of the mean result

We used our RA model to obtain the elevated levels of cellular TM and sTM, when the cells were treated with a physiological concentration of RA, RA_plasma_ = 5 nM [46]. Treating the combination of the gene expression model and the sTM model with 5 nM of RA resulted in a 9% increase in the mean cellular TM level and subsequently the mean plasma level of sTM. We then investigated the effects of physiological concentrations of RA on the thrombin generation profile (Fig. 15). The absence of vitamin A in the diet increased the peak level of thrombin up to 10%, while this increase was up to 11% for the ETP.

## Discussion

Clinical observations have shown that RA has therapeutic effects on blood coagulation disorders such as DIC and thrombosis in cancer patients. Previous studies have mainly looked at RA as a treatment for cancer. Corrective effects of RA on coagulation disorders, which is a positive side effect of RA therapy, have rarely been studied. Elucidating the mechanism of action of RA in the treatment of coagulation disorders is important, since this can help to understand why some patients respond to the drug better than others. This is also useful in developing new drugs with better therapeutic effects. Down regulation of TF and CP on cancer cells and monocytes, upregulation of u-PA, PAI-1 and PAI-2 in cancer cells, and upregulation of TM and t-PA in endothelial cells have been introduced as the possible mechanisms for RA to ameliorate blood coagulation disorders. In this study, we investigated how RA therapy regulates TM expression and how this variation affects the thrombin generation profile. We developed a gene expression model for the RA-induced upregulation of TM. Coupling the gene expression model with a two-compartment pharmacokinetic model of RA, we simulated the time-dependent variations of TM and TMR concentrations after taking different oral doses of RA. Our results indicate that the TM concentration increases almost twofold after taking a 110 $\frac{mg}{m^2}$ oral dose of RA. Since most of the patients who are under RA treatment take RA on a daily basis, TM expression on the endothelial cell surface changes over time. Our results indicate that RA treatment increases the mean value of TM concentration, while the nature of this regulation is oscillatory. To examine the effects of the upregulated TM on the blood coagulation system, we used a mathematical model for the human coagulation cascade [39]. Our simulations show that taking a 45 $\frac{mg}{m^2}$ or 110 $\frac{mg}{m^2}$ oral dose of RA reduces ETP by 45 and 49%, respectively. Furthermore, our results are useful in predicting the times when a patient is at a higher risk of clot formation. Almost 14 hours after drug ingestion, the TM concentration begins to decrease, and reaches its minimum level almost before taking the next dose of the drug. However, the minimum level of TM during the RA treatment period is higher than the normal level of TM when there is no RA treatment. We also investigated the effects of progressive reductions in the plasma concentration of RA over a course of continuous treatment on thrombin generation. Our results indicated that the progressive reductions in plasma concentration of RA over the course of RA therapy with daily oral dosing (110 $\frac{mg}{m^2}$), which has been observed in some cancer patients, do not affect the corrective effects of RA therapy on thrombin generation significantly. These results prompt the hypothesis that coagulation abnormalities may become resistance later than cancer to RA. The validity of this hypothesis depends on various patient- and cancer-specific factors such as the RA route of administration, the adequate plasma concentration of RA for cancer treatment, the acceptable range of reduction in plasma RA over the course of RA therapy, and the allowable range of toxicity. Thus, the validity of this hypothesis should be tested for different groups of patients with various cancer types, stages and health conditions such as liver and kidney health, independently. Taken together, our simulations indicate that oscillatory expression of TM over the course of RA therapy can play a critical role in the regulation of thrombin production. This finding may explain why RA therapy improves DIC and thrombosis in some cancer patients better than in others. Our simulation results suggest that one possible reason might be the impairment of PC pathway because of cancer, cancer treatment, etc. It is important to note that the current study cannot compare the significance of TM with other potentially important proteins such as t-PA, u-PA, PAI-1, PAI-2, TF and CP, in RA-induced improvement of clotting disorders in cancer patients. Further experimental and numerical studies are needed to investigate the contributions of the above pathways to RA therapy of DIC in cancer. This study can be considered as a starting point for research studies exploring the possible effects of oscillatory protein expression after drug administration, on the blood coagulation cascade.

There are some limitations to this study. First, we simulated the thrombin generation process using the physiological levels of all blood factors except TF and TM. This is because some cancer patients have coagulation factor levels within the normal ranges [58, 59]. However, this is not true for all cancer patients. Plasma concentrations of coagulation factors in cancer patients depend on several factors such as type and stage of the cancer, and type of the antitumor therapy. Thus, the quantitative aspect of our results on corrective effects of RA therapy on thrombin generation, cannot be applied to all patients with different disease conditions. Second, we did not consider the effects of RA treatment on the cancer cells’ ability to produce inflammatory cytokines. Previous in vitro studies have indicated that RA treatment increased the production of some inflammatory cytokines such as IL-1β by cancer cells [60, 61]. In theory, induction of cytokine release can favor the prothrombotic potential of the endothelium by upregulating TF and downregulating TM expression [62, 63]. However, we believe that the elevated levels of plasma cytokines after treatment with the specified doses of RA in this study should not influence the TF and TM expression significantly [60, 62, 64]. Third, the model we used for simulating thrombin generation was developed based on in vitro assays. Even though all coagulation factor concentrations were physiological concentrations, the model cannot capture some essential features of the coagulation in vivo, such as the cellular involvement and the effects of flow. In fact, our model cannot describe exactly how RA therapy improves clotting disorders in vivo. Instead, our simulation results indicate that the oscillatory variation in TM expression over the course of RA therapy significantly influenced in vitro thrombin generation. In vivo studies are needed to confirm the key role of TM in RA treatment of coagulopathy. Fourth, in constructing the sTM release model, for simplicity we assumed that the cellular TM is degraded by a first-order reaction. The kinetic order of the reaction depends upon the types and concentrations of degradation enzymes and the severity of cell injury. However, little is known about the types and concentrations of the enzymes which are primarily responsible for producing sTM from cellular TM. Furthermore, the severity of cell injury depends on the type and stage of the cancer. Thus, the model presented in this section cannot describe TM cleavage for all cancer patients with different conditions. The model can be improved once more information about the degradation pathway is available. We have also assumed that the amount of cellular TM is not significantly reduced due to release of the TM into the plasma. This is because the number of sTM molecules in plasma is much smaller than the number of TM molecules on the endothelium under physiological conditions. Next, we assumed that the unbound fraction of RA in plasma was constant over the course of RA treatment. However, the unbound fraction of RA depends on different variables, such as the serum albumin concentration in plasma, the total concentration of RA in plasma, and the levels of other drugs in blood. Cancer patients usually take different medications at the same time. Furthermore, the serum albumin level can be affected by cancer and cancer treatment. Thus, the exact quantitative effects of RA therapy on TM expression can vary from patient to patient.

## Conclusions

All-trans retinoic acid (RA) has been widely used to treat various types of cancer. RA treatment also improves coagulation abnormalities in cancer patients. However, it is not clear how RA therapy ameliorates coagulation disorders. In this study, for the first time, we developed a mechanistic model to investigate the role of thrombomodulin (TM) in RA therapy of cancer-induced coagulation disorders. Our results indicate that RA-induced TM upregulation reduces thrombin generation significantly. Daily administration of the drug results in oscillatory expression of TM on endothelial cells. We also demonstrate that within 2 weeks of continuous RA treatment, TM expression patterns remain almost unchanged, while some cancers become resistant to RA therapy. This result raises the hypothesis that RA therapy has longer lasting corrective effects on coagulation disorders than on cancer. Clinical studies and in vivo experiments are required to test the validity of this hypothesis. Overall, our findings indicate the key role of TM in RA treatment of blood coagulation abnormalities in cancer patients. These results are in line with recent clinical observations regarding the therapeutic effects of recombinant human thrombomodulin, an anticoagulant drug with the same external structure of TM, in the treatment of DIC [65–69].

Moving forward, we plan to couple this model with other mechanistic models that simulate the effects of RA therapy on the expression levels of TF, CP, PAI-1, PAI-2, t-PA and u-PA, and compare the significance of different pathways in RA therapy of clotting disorders in cancer patients. Such models should be able to simulate how the RA treatment downregulates the expression of TF and CP and upregulates the synthesis of PAI-1, PAI-2, t-PA and u-PA in various cell types such as endothelial cells, monocytes, and tumor cells. Mechanistic modeling of these pathways requires concurrent experimental studies to explore the relevant biological pathways.

## Additional file

Additional file 1: Detailed model description and supplementary results. (DOCX 330 kb)

## Abbreviations

APL: Acute promyelocytic leukemia
CP: Cancer procoagulant
DIC: Disseminated intravascular coagulation
ETP: Endogenous thrombin profile
ODE: Ordinary differential equation
PAI-1: Plasminogen activator inhibitor 1
PAI-2: Plasminogen activator inhibitor 2
PC: Protein C
RA: All-trans retinoic acid
sTM: Soluble TM
TM: Thrombomodulin
TMR: Thrombomodulin-mRNA
t-PA: Tissue plasminogen activator
u-PA: Urokinase plasminogen activator
