# Supplementary material for: Analysis of the role of thrombomodulin in all-trans retinoic acid treatment of coagulation disorders in cancer patients
Source: Theor Biol Med Model. 2019 Feb 14;16:3. doi: 10.1186/s12976-019-0099-z (PMC6376718; doi:10.1186/s12976-019-0099-z)
Supplement: Supplementary file 1 — Detailed model description and supplementary results. (DOCX 330 kb) [file 12976_2019_99_MOESM1_ESM.docx]

**Additional file 1**

1. **All-trans retinoic acid pathway**

RA is highly bound to albumin in plasma [1] and in the cell culture medium [2]. Free RA molecules start to passively diffuse across the cellular membrane once the cultured cells are treated by RA. Passive diffusion continues to occur until the concentration of free RA on both sides of the cellular membrane are equal. Equilibrium is achieved relatively quickly due to the high permeability coefficient of RA across the endothelial cell membrane [3] and high surface area per volume of endothelial cells. After diffusing across the membrane, RA molecules bind to different receptors (Fig. S1). Some of the RA binding receptors can initiate the transcription of target genes after binding to RA. When the transcription factors bind to RA molecules, they become activated and trigger the transcription of the TM gene by binding to DNA at a retinoic acid response element (RARE) located in enhancer regions of the gene [4, 5].


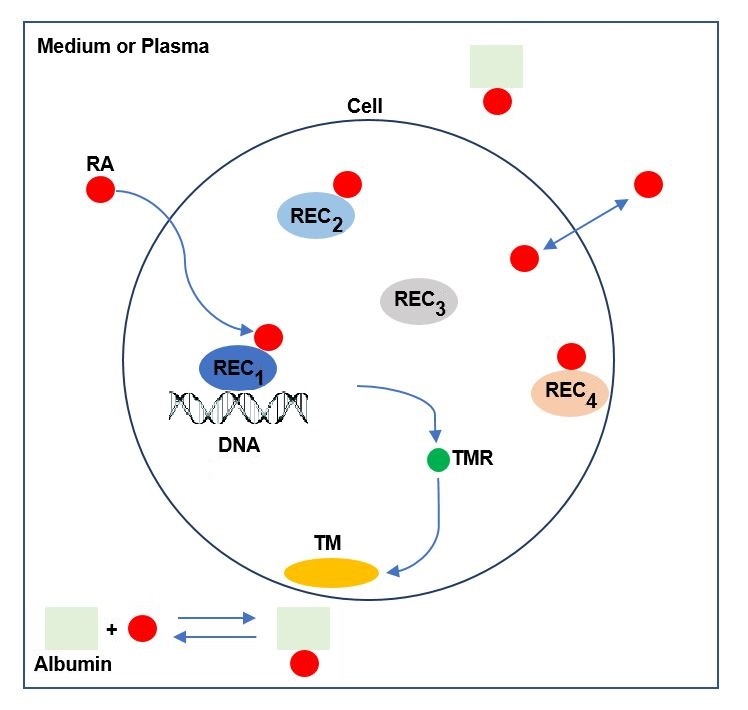


Figure S1. Simplified schematic of the RA signaling pathway. RA is mainly bound to albumin outside of the cells. RA molecules (red circles) can diffuse across the cellular membrane and bind to different receptors ($\mathrm{REC}_{1}$, $\mathrm{REC}_{2}$, $\mathrm{REC}_{3}$, etc). Some of the RA binding receptors ($\mathrm{REC}_{1}$, in this cartoon diagram) can initiate the transcription of the TM gene after binding to RA molecules.

The binding of RA to a single site on a RA binding receptor ($\mathrm{REC}$) is expressed by

$$RA+REC\overset{\Leftrightarrow}{k_{d1}}RA:REC ,$$

where $k_{d1}$ is the equilibrium dissociation constant,

$$k_{d1}=\frac{\left[ \mathrm{RA} \right][REC]}{\left[ RA:REC \right]} , (S1)$$

and [] indicates molar concentration. Thus, $\left[ \mathrm{RA} \right]$, $[REC]$ and $\left[ RA:REC \right]$ represent molar concentrations of free RA, free RA binding receptor and bound receptor ($RA:REC$), respectively. Assuming that RA is either free in the cell or bound to the RA binding receptors ($\mathrm{REC}$), Eq. S1 can be written as

$$k_{d1}=\frac{\left[ \mathrm{RA} \right](\left[ \mathrm{REC}_{t} \right]-[RA:REC])}{[RA:REC]} , (S2)$$

where $[\mathrm{REC}_{t}]$ stands for molar concentration of all RA binding receptors,

$${[REC}_{t}]={[REC}_{1}]+{[REC}_{2}]+\ldots(S3)$$

Solving Eq. S2 for the bound fraction of RA binding receptors yields

$$\frac{[RA:REC]}{{[REC}_{t}]}=\frac{[\mathrm{RA}]}{\left[ \mathrm{RA} \right]+k_{d1}} . (S4)$$

The concentration of the specific transcription factor ($\mathrm{REC}_{1}$) which can activate the transcription of the TM gene is a portion of the total concentration of RA binding receptors ($\mathrm{REC}_{t}$). Assuming that RA has the same affinity to bind to different RA binding receptors, the ratio of the activated transcription factor concentration ([$RA:\mathrm{REC}_{1}]$) to the total transcription factor concentration ($[\mathrm{REC}_{1t}$]) is the same as the ratio of the activated RA receptors concentration ($[RA:REC]$) to the total RA receptors concentration ($[\mathrm{REC}_{t}]$), thus

$$\frac{[RA:REC]}{[\mathrm{REC}_{t}]}=\frac{[{RA:REC}_{1}]}{[\mathrm{REC}_{1t}]} . (S5)$$

The assumption that RA binds to various types of RA binding receptors with the same affinity is reasonable, since the majority of RA binding receptors found in endothelial cells are from the nuclear hormone receptor family. The retinoic acid receptors ($\mathrm{RAR}_{\alpha}$,$\mathrm{RAR}_{\beta}$,$\mathrm{RAR}_{\gamma}$) and retinoid X receptors ($\mathrm{RXR}_{\alpha}$,$\mathrm{RXR}_{\beta}$,$\mathrm{RXR}_{\gamma}$) are the main members of the nuclear hormone receptor family.

The transcription rate of the TM gene depends on the promoter occupancy. The binding of an activated transcription factor to a promoter can be described by

$$RA:\mathrm{REC}_{1}+Promoter\overset{\Leftrightarrow}{k_{d2}}RA:\mathrm{REC}_{1}:Promoter ,$$

where $k_{d2}$ is the equilibrium dissociation constant of the transcription factor binding to the promoter. The fraction of time that any given promoter spends in the transcription factor-bound state is given by [6-8]

$$Fraction of binding time=\frac{[RA:\mathrm{REC}_{1}]}{[RA:\mathrm{REC}_{1}]+k_{d2}} . (S6)$$

The rate of the gene transcription is proportional to the fraction of the binding time,

$$\frac{I}{I_{\max}}=\frac{[RA:\mathrm{REC}_{1}]}{\left[ RA:\mathrm{REC}_{1} \right]+k_{d2}} , (S7)$$

where $I_{\max}$is the maximum transcription rate by a specific transcription factor ($\mathrm{REC}_{1}$).

The transcription rate ($I$) can be obtained as a function of $RA,$ $\mathrm{REC}_{1t}$, and $I_{\max}$ by combining Eqs. S4, S5 and S7,

$$I=I_{\max}\frac{\left[ \mathrm{RA} \right][\mathrm{REC}_{1t}]}{\left[ \mathrm{RA} \right][\mathrm{REC}_{1t}]+k_{d2}([RA]+k_{d1})} . (S8)$$

It is important to note that we assumed the association/dissociation event between RA and REC, and the binding/unbinding of $RA:\mathrm{REC}_{1}$ to promoter are in equilibrium. This is because these reactions occur on much faster time scales than gene transcription [9-11]. Furthermore, DNA-transcription factor reactions are much faster than protein-ligand reactions [10, 12].

1. **Estimation of gene expression model parameters**
   1. **Parameter estimation algorithm**

The gene expression model had four species, i.e. TMR, TM, RA and $\mathrm{REC}_{1t}$, and seven parameters, namely $I_{0},$ $k_{\mathrm{trans}}$, $I_{\max}$, $k_{\mathrm{dp}},$ $k_{\mathrm{dm}},$ $k_{d1}$and $k_{d2}$. The model parameters and species were defined in Table 1. The values of $k_{\mathrm{dp}},$ $k_{\mathrm{dm}}$, $k_{d1}$ and $k_{d2}$ were taken from the literature (Table 1), while the values of the remaining parameters, and the initial concentrations of the model species, i.e. ${IC}_{\mathrm{TM}}, {IC}_{\mathrm{TMR}}$ and $[\mathrm{REC}_{1t}]$ were unknown. $k_{\mathrm{trans}}$ and basal transcription rate ($I_{0})$ were the only parameters that depended on the other parameters. Assuming that the TM and TMR concentrations were in steady state before RA treatment (t=0), we calculated $k_{\mathrm{trans}}$ and $I_{0}$ by

$$k_{\mathrm{trans}}=\frac{k_{\mathrm{dp}}{IC}_{\mathrm{TM}}}{\mathrm{IC}_{\mathrm{TMR}}}, (S9)$$

$I_{0}=k_{\mathrm{dm}}{IC}_{\mathrm{TMR}} .$ $(S10)$

Overall, the gene expression model had three independent unknown parameters namely, $I_{\max}$, ${IC}_{\mathrm{TMR}}$, $[\mathrm{REC}_{1t}]$, and two dependent parameters, namely $k_{\mathrm{trans}}$ and $I_{0}$. We instituted some bounds for all model unknown parameters (Table 1). The bounds for ${IC}_{\mathrm{TM}}$ were due to the experimental errors (Fig. 2), while the other unknown parameters had physiological bounds. The gene expression model unknown parameters were estimated by minimizing the squared difference between simulation results and the experimental data following a parameter estimation algorithm. The parameter estimation process was designed in such a way that the simulation results for the TMR concentration at different time points, i.e. 0, 3, 6, 12, 24 h, when RA =10 µM and the simulation results for TM concentration at different RA concentrations, i.e. 0.1, 1, 10 µM, when t= 24 h fit the experimental data shown in Fig 2. The objective function was defined by

$$E=\sum_{i=1}^{5} ({M_{i}^{s}-{[TMR]}_{i}^{s})}^{2}+\sum_{j=1}^{3} {(1-{[TM]}_{j}^{s})}^{2}. (S11)$$

The first term in the objective function quantified the difference between the scaled experimental TMR concentration ($M_{i}^{s}$) and scaled numerical results for TMR level (${[TMR]}_{i}^{s}$) at time point i. Time points i=1, 2, 3, 4 and 5 stand for t=0, 3, 6, 12 and 24, respectively, while the superscript s shows that the values are scaled. Scaled experimental TMR concentration and scaled numerical TMR levels were given by

$$M_{i}^{s}=\frac{M_{i}-M_{1}}{M_{5}-M_{1}} (S12)$$

$${[TMR]}_{i}^{s}=\frac{{[TMR]}_{i}-{[TMR]}_{1}}{{[TMR]}_{5}-{[TMR]}_{1}} (S13)$$

where the indices i=1 and 5 correspond to t=0 and 24 h, respectively. The values of $M_{i}$ were shown in Fig. 2b, while $[TMR]$ was the output of gene expression model. It is important to note that the experimental results presented in Fig. 2b were normalized by TMR concentration before RA treatment. However, under the new scaling (Eq. S12), the lowest TMR concentration in the data set was 0, while the largest TMR concentration was 1 (0 $\leq$ $M_{i}^{s}\leq1$).

The second term in Eq. S11 represented the sum of squared differences between the scaled numerical results and the experimental observations for TM concentration at t= 24h for different RA concentrations, i.e. 0.1 µM, 1µM and 10 µM. Different values of index j in Eq. S11 represent different RA concentrations; j=1, 2 and 3 represent RA=0.1, 1 and 10 µM, respectively. The scaled numerical TM level (${[TM]}_{j}^{s})$ was defined as the TM concentration from the model divided by experimental observation

$${[TM]}_{j}^{s}=\frac{{[TM]}_{j}^{t=24}}{N_{j}^{t=24}} (S14)$$

where $[TM]$ and N denote simulation results and experimental data for TM concentration, respectively. Subscript j denotes RA concentration, while superscript (t=24) indicates that the values are for 24 hours after RA treatment. The ranges of variation of $N_{i}$ were shown in Fig. 2a, while $[TM]$ was the output of gene expression model.

The parameter estimation process was initiated by determining TM initial concentration (${IC}_{\mathrm{TM}}$) at various concentrations of RA. The experimental measurements for the TM concentration contained error bars (Fig. 2a). However, we needed to have constant values for the TM concentration at the different concentrations of RA. Thus, we randomly selected values for the TM concentration at the various concentrations of RA following a normal distribution. The bars in Fig. 2a denote the mean values of the TM level at various concentrations of RA, while the standard deviation is half the length of the total error bar. Using Particle swarm optimization (PSO), a population-based stochastic optimization technique, we estimated the independent unknown parameters, i.e. $I_{\max},$ ${IC}_{\mathrm{TMR}}$ and $[\mathrm{REC}_{1t}]$ in a way such that the simulation results for TMR and TM levels fit the experimental data for various concentrations of RA (Fig. 3). After estimating the unknown parameters, we calculated the dependent parameters, i.e. translation rate constant and basal transcription rate via Eq. 4 and Eq. 5, respectively. If the calculated values were within the physiological ranges shown in Table 1, the obtained set of the parameters was accepted. Otherwise, we took a step back and chose a new set of values for the TM concentration at the various concentrations of RA, and the same procedure for fitting the parameters was repeated. Figure S2 outlines the implemented procedure for model parameter estimation.


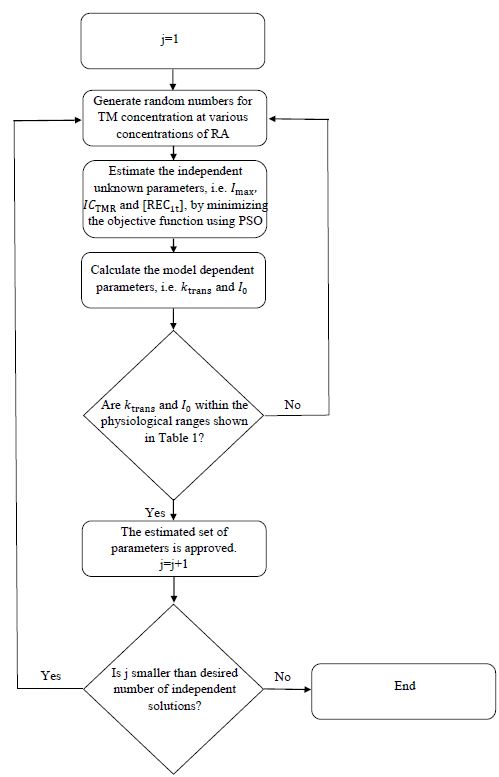


Figure S2. Flow chart of the parameter estimation algorithm.

It is important to note that our problem does not have a unique solution, since the experimental data used for fitting the model parameters is subject to error (Fig. 2a). The estimated values for the unknown parameters depend on the randomly selected values for the TM concentration at RA=10 μM, RA= 1 μM, and RA=0.1 μM. Thus, we repeated the parameter estimation algorithm 150 times independently and obtained several sets of valid solutions.

The obtained values for ${IC}_{\mathrm{TMR}}$, $I_{max}$ and $[\mathrm{REC}_{1t}]$ were within the ranges shown in Fig. S3. The y-axis range in Fig. S3 shows the physiological range for each parameter.


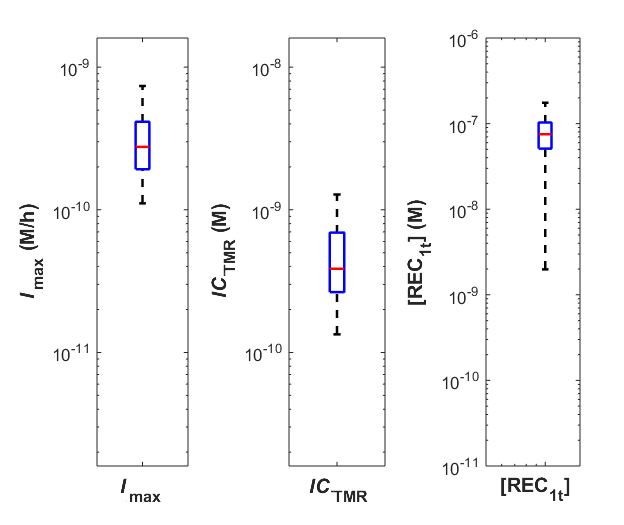


Figure S3. Ranges of the obtained values for the TM transcription rate, the TMR initial concentration, and the total concentration of the transcription factor that activates TM gene. These results were based on 150 valid solutions.

The mean estimated value for total transcription factor concentration were between $7\times{10}^{-8}M$ (Fig. S3), which is comparable with the reported value of $5\times{10}^{-8}M$ for RAR concentration in promyelocytic leukemia cells [13]. The obtained values for dependent unknown parameters, i.e. $k_{\mathrm{trans}}$ and $I_{0}$were within the ranges shown in Fig. S4. The y-axis range in Fig. S4 shows the physiological range for each parameter.


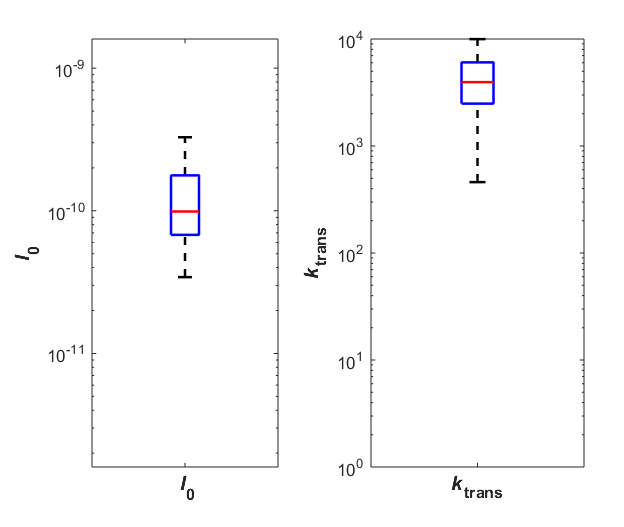


Figure S4. Ranges of the obtained values for the translation rate constant and the basal transcription rate.

- 1. **Particle swarm optimization**

Particle swarm optimization (PSO) method has been shown to be efficient in estimating ODE model parameters [14, 15]. PSO involves a swarm of particles, where each particle represents a point in a D-dimensional space, where D is the number of parameters to be estimated. PSO finds the global optimum of the objective function by iteratively changing the positions of the particles. In PSO, the position of each particle in the parameter space is changed based on the experience, or knowledge, of the particle and its neighbors. Suppose that particles are randomly distributed in the parameter space at t=0, and the position of the ith particle in a D dimensional parameter space can be described by the vector

$\boldsymbol{x}_{i}=\left[ x_{i1}, x_{i2},\ldots, x_{\mathrm{iD}} \right]. (S15)$

PSO calculates the objective function value (aka fitness value) for each particle and iteratively updates the position of each particle by

$$\boldsymbol{x}_{i}\left( t+1 \right)=\boldsymbol{x}_{i}\left( t \right)+\boldsymbol{v}_{i}\left( t+1 \right) (S16)$$

where $t$ and $t+1$ are two consecutive iterations of the algorithm, and $\boldsymbol{v}_{i}$ indicates the vector of velocity components of the ith particle in the D-dimensional parameter space. The velocity of the ith particle is defined by

$$\boldsymbol{v}_{i}\left( t+1 \right)=\boldsymbol{v}_{i}\left( t \right)+\theta_{1}\left( p_{i}-\boldsymbol{x}_{i}\left( t \right) \right)R_{1}+\theta_{2}\left( g-\boldsymbol{x}_{i}\left( t \right) \right)R_{2}, (S17)$$

where $p_{i}$ and g denote the local best solution found by the ith particle and the best solution found over the entire population of the particles. $\theta_{1}$ and $\theta_{2}$, which are cognitive and social coefficients, respectively, modulate the magnitude of the steps taken by the particle. In this study, we used ($\theta_{1}$, $\theta_{2}$)=(0.05564, 0.02886), [14]. $R_{1}$ and $R_{2}$are random vectors generated from a uniform distribution in [0,1]. The first term in Eq. S17 is called the inertia component and prevents the particle from significantly changing direction, while the second term in Eq. S17 is called the cognitive component, which indicates that the particles prefer to return to their own previously found best positions. The third term in Eq. S17 is named the social component, which accounts for the tendency of the particles to move towards the position of the particle which has the lowest objective function value. After updating the particle positions at each time step, PSO calculates the objective function value for all particles. PSO then updates the personal best position for each particle and the global best position over the whole population of the particles. This iterative optimization continues until a stopping criterion is met. The global best particle is represented as the best solution at the end of optimization process. Figure S5 shows the flow diagram of the PSO. In this study, the population size (number of particles) was 1000, while the number of generations (Gen) was 100.


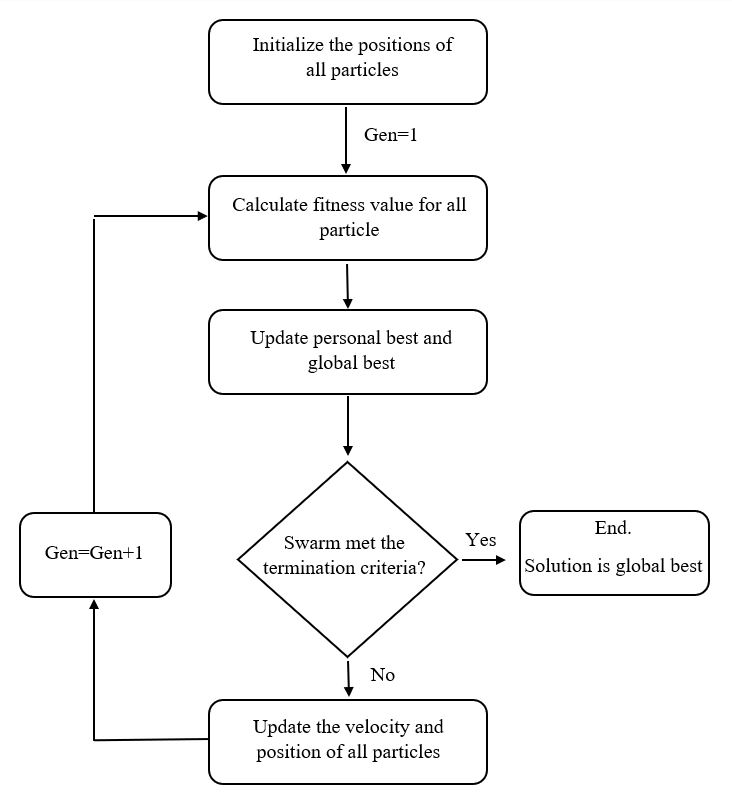


Figure S5. Flow chart of the particle swarm optimization algorithm.

1. **Population pharmacokinetic modelling of RA**

We constructed a two-compartment pharmacokinetic model (Fig. 6) in MATLAB SimBiology and fit the model parameters, i.e. $k_{a}, k_{d}$, $k_{\mathrm{cp}}$, $k_{\mathrm{pc}},$ $V_{c}$ and $V_{p}$ to the data shown in Fig. 5. $k_{\mathrm{cp}}$ and $k_{\mathrm{pc}}$ were the distribution rate constant from the central compartment to the peripheral compartment, and the distribution rate constant from the peripheral compartment to the central compartment, respectively. $k_{\mathrm{cp}}$ and $k_{\mathrm{pc}}$ were defined by

$$k_{\mathrm{cp}}=\frac{Q_{\mathrm{cp}}}{V_{c}} (S18)$$

$$k_{\mathrm{pc}}=\frac{Q_{\mathrm{cp}}}{V_{p}}, (S19)$$

where $Q_{\mathrm{cp}}$ is the intercompartmental clearance [16]. We used a MATLAB function called nlmefista, which is designed to find the maximum likelihood estimates of the parameters by fitting a nonlinear mixed-effects regression model. Nonlinear mixed-effects regression models have been widely used for the analysis of pharmacokinetic data, as they can consider inter-subject variability in parameters by incorporating random effects into the model [17]. The estimated values for $k_{a}, k_{d}$, $Q_{\mathrm{cp}},$ $V_{c}$ and $V_{p}$ were 0.62 1/h, 0.56 1/h, 0.011 L/h , 81.92 L and 3.01 L, respectively. The simulation results corresponding to these estimated values were shown by solid lines in Fig. 5. We used other fitting methods in MATLAB SimBiology such as fminsearch and lsqcurvefit which are used to fit a model to data using a derivative free method [18] and a nonlinear least-square curve fitting technique [19]. The curves fit by different methods looked very similar. The estimated values for $k_{a}, k_{d}$ and $V_{c}$ by different methods were very close, while the estimated values for $Q_{\mathrm{cp}}$ and $V_{p}$ depended strongly on the fitting method. Thus, $Q_{\mathrm{cp}}$ and $V_{p}$ were not identifiable considering the size of the data set. Additional data points were needed to obtain more accurate estimates for the parameter. However, the main goal of this study was not estimating the pharmacokinetic parameters following oral administration of RA. We aimed to investigate the effects of RA therapy on thrombin generation by coupling four different models, i.e. the pharmacokinetic model, the gene expression model, the sTM release model and the ODE model of the coagulation cascade. Since the curves fit to the pharmacokinetic data through different methods were almost the same, we used the output of the pharmacokinetic model, i.e. fitted curves, as input in the gene expression model to investigate the variations of TM and TMR following RA therapy. Pharmacokinetic parameters of RA following oral dosing can be better estimated using the presented model in this paper once there is more clinical data available.

**References**

1 Thatcher, J. E., Zelter, A., Isoherranen, N. 2010 The relative importance of CYP26A1 in hepatic clearance of all-trans retinoic acid. *Biochemical pharmacology*. **80**, 903-912.

2 Horie, S., Ishii, H., Matsumoto, F., Kusano, M., Kizaki, K., Matsuda, J., Kazama, M. 2001 Acceleration of Thrombomodulin Gene Transcription by Retinoic Acid RETINOIC ACID RECEPTORS AND Sp1 REGULATE THE PROMOTER ACTIVITY THROUGH INTERACTIONS WITH TWO DIFFERENT SEQUENCES IN THE 5′-FLANKING REGION OF HUMAN GENE. *Journal of Biological Chemistry*. **276**, 2440-2450.

3 Franke, H., Galla, H.-J., Beuckmann, C. T. 2000 Primary cultures of brain microvessel endothelial cells: a valid and flexible model to study drug transport through the blood–brain barrier in vitro. *Brain Research Protocols*. **5**, 248-256.

4 Duester, G. 2008 Retinoic acid synthesis and signaling during early organogenesis. *Cell*. **134**, 921-931.

5 Marchetti, M., Vignoli, A., Bani, M. R., Balducci, D., Barbui, T., Falanga, A. 2003 All-trans retinoic acid modulates microvascular endothelial cell hemostatic properties. *Haematologica*. **88**, 895-905.

6 Ingalls, B. 2013 Mathematical modelling in systems biology: An introduction. *Internet.[cited at p. 117]*.

7 Sauro, H. M. 2011 *Enzyme kinetics for systems biology*. Future Skill Software.

8 Stormo, G. D., Zhao, Y. 2010 Determining the specificity of protein–DNA interactions. *Nature Reviews Genetics*. **11**, 751-760.

9 Ingalls, B. P. 2013 *Mathematical modeling in systems biology: an introduction*. MIT press.

10 Halford, S. E., Marko, J. F. 2004 How do site‐specific DNA‐binding proteins find their targets? *Nucleic acids research*. **32**, 3040-3052.

11 Crettaz, M., Baron, A., Siegenthaler, G., Hunziker, W. 1990 Ligand specificities of recombinant retinoic acid receptors RAR α and RAR β. *Biochemical journal*. **272**, 391-397.

12 Mirny, L., Slutsky, M., Wunderlich, Z., Tafvizi, A., Leith, J., Kosmrlj, A. 2009 How a protein searches for its site on DNA: the mechanism of facilitated diffusion. *Journal of Physics A: Mathematical and Theoretical*. **42**, 434013.

13 Nervi, C., Grippo, J. F., Sherman, M. I., George, M. D., Jetten, A. M. 1989 Identification and characterization of nuclear retinoic acid-binding activity in human myeloblastic leukemia HL-60 cells. *Proceedings of the National Academy of Sciences*. **86**, 5854-5858.

14 Sagar, A., Varner, J. D. 2015 Dynamic modeling of the human coagulation cascade using reduced order effective kinetic models. *Processes*. **3**, 178-203.

15 Sagar, A., Dai, W., Minot, M., LeCover, R., Varner, J. D. 2017 Reduced order modeling and analysis of the human complement system. *PloS one*. **12**, e0187373.

16 Hill, S. 2004 Pharmacokinetics of drug infusions. *Continuing education in anaesthesia, critical care & Pain*. **4**, 76-80.

17 Drikvandi, R. 2017 Nonlinear mixed-effects models for pharmacokinetic data analysis: assessment of the random-effects distribution. *Journal of pharmacokinetics and pharmacodynamics*. **44**, 223-232.

18 Rios, L. M., Sahinidis, N. V. 2013 Derivative-free optimization: a review of algorithms and comparison of software implementations. *Journal of Global Optimization*. **56**, 1247-1293.

19 Griva, I., Nash, S. G., Sofer, A. 2009 *Linear and nonlinear optimization*. Siam.
